# Immune-Mediated Competition in Rodent Malaria Is Most Likely Caused by Induced Changes in Innate Immune Clearance of Merozoites

**DOI:** 10.1371/journal.pcbi.1003416

**Published:** 2014-01-23

**Authors:** Jayanthi Santhanam, Lars Råberg, Andrew F. Read, Nicholas Jon Savill

**Affiliations:** 1 Institute of Immunology and Infection Research, University of Edinburgh, Ashworth Labs, Edinburgh, Scotland; 2 Department of Biology, Lund University, Lund, Sweden; 3 Center for Infectious Disease Dynamics, The Pennsylvania State University, University Park, Pennsylvania, United States of America; University of Michigan and Howard Hughes Med. Inst., United States of America

## Abstract

Malarial infections are often genetically diverse, leading to competitive interactions between parasites. A quantitative understanding of the competition between strains is essential to understand a wide range of issues, including the evolution of virulence and drug resistance. In this study, we use dynamical-model based Bayesian inference to investigate the cause of competitive suppression of an avirulent clone of *Plasmodium chabaudi* (AS) by a virulent clone (AJ) in immuno-deficient and competent mice. We test whether competitive suppression is caused by clone-specific differences in one or more of the following processes: adaptive immune clearance of merozoites and parasitised red blood cells (RBCs), background loss of merozoites and parasitised RBCs, RBC age preference, RBC infection rate, burst size, and within-RBC interference. These processes were parameterised in dynamical mathematical models and fitted to experimental data. We found that just one parameter 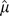, the ratio of background loss rate of merozoites to invasion rate of mature RBCs, needed to be clone-specific to predict the data. Interestingly, 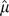 was found to be the same for both clones in single-clone infections, but different between the clones in mixed infections. The size of this difference was largest in immuno-competent mice and smallest in immuno-deficient mice. This explains why competitive suppression was alleviated in immuno-deficient mice. We found that competitive suppression acts early in infection, even before the day of peak parasitaemia. These results lead us to argue that the innate immune response clearing merozoites is the most likely, but not necessarily the only, mediator of competitive interactions between virulent and avirulent clones. Moreover, in mixed infections we predict there to be an interaction between the clones and the innate immune response which induces changes in the strength of its clearance of merozoites. What this interaction is unknown, but future refinement of the model, challenged with other datasets, may lead to its discovery.

## Author Summary

Malaria infections often consist of more than one strain of the same parasitic species. Understanding the within-host competition between these various strains is essential to understanding the evolution and epidemiology of drug resistance in malarial infections. The infection process and the competition between strains involve complicated biological processes that are explained by various hypotheses. Mathematical models tested against experimental data provide quantitative measures to compare these hypotheses and enable us to discern the actual biological processes that contribute to the observed dynamics. We use a group of models against experimental data on rodent malaria to test various hypotheses. Such quantitative measures, in understanding rodent malaria, can be considered as a step towards understanding within-host parasite dynamics. Our work presented here demonstrates how confronting mathematical models with data allows the discovery of subtle and novel interactions between hosts and parasites that would be impractical to do in an experiment and allows the rejection of hypotheses that are incorrect. It is our contention that understanding the forces controlling within-host parasite dynamics in well-defined experimental model is a necessary step towards understanding these features in natural infections.

## Introduction

Malarial infections often consist of more than one strain of the same parasitic species [1]–[3]. Parasite populations of multiple strains interact with one another directly via resource competition and indirectly via the host's immune response to the infection [4], [5]. These interactions affect the population dynamics of the competing strains [2], [4]–[13]. Population dynamics during such mixed infections, when compared to single infections, have been shown to exhibit different mortality rates for the parasites, rates of growth to peak density, maximum parasitaemia and renewed growth within hosts [6]. There is evidence to suggest that higher within-host densities may lead to higher transmission success [7], [14] and competitive interactions which may directly affect the rate of transmission [3], [8]. Such competitive interaction can drive the evolution of virulence in parasites [7], [15]. Consequently, understanding the within-host competition between strains, is essential to understanding the evolution of virulence and drug and vaccine resistance in malarial infections [7], [16]–[18].

Several experimental studies of mixed infections of *P. chabaudi* clones have demonstrated competitive suppression of less virulent clones by virulent clones [7], [14], [15], [19]. These studies have led to some interesting speculation on the potential mechanisms responsible for the competitive suppression. However, an exact mechanism has yet to be established. An experimental study of mixed infections of two *P. chabaudi* clones, by Taylor et al., provides evidence for competitive suppression of one of the clones irrespective of initial dose [14]. Mice in three treatment groups were infected with virulent (ER) and avirulent (CR) clones of *P. chabaudi* with different ratios of initial parasite numbers. The competitive suppression of avirulent clone at the later stages of infection in all three treatment groups were attributed to clone-specific and cross-immunity of the host induced by the parasite strains. However, the exact role of host immune response on the suppression of CR could not be explored.

In another experimental study, 7 genetically closely related strains of *P. chabaudi*, differing in virulence, were tested against an unrelated, and more virulent strain of *P. chabaudi* [7]. Densities of individual parasite strains in mixed infections were tracked for 14–50 days. In all infections the virulent strain competitively suppressed the avirulent strains. Among the avirulent strains, the ones that were more virulent in single-strain infections achieved greater parasite densities and also suffered relatively less competitive suppression than the less virulent strains when in competition. The exact mechanism by which the avirulent clone is suppressed could not be established.

Another study showed that a virulent clone obtained a competitive advantage due to larger parasite and gametocyte densities, compared to an avirulent clone, during mixed infections [15]. Compared to respective single infections, both strains experienced reduction in both asexual parasite and gametocytes densities. However, the suppression in the gametocytes density of an avirulent clone was larger compared to the virulent strain during mixed infections. Virulent clones reached larger parasite densities compared to avirulent clones both in single and mixed infections. This study demonstrated the importance of within-host competition in the spread and selection of virulence in parasite evolution.

Recently a series of experiments were designed to study the effects of parasite genotype, residency and time of infection on within-host parasite densities during mixed infections. In these experiments two pairs of distinct clones of *P. chabaudi* were inoculated into mice either simultaneously or 3 or 11 days apart and their population sizes were tracked using immunofluorescence or quantitative polymerase chain reaction [19]. In all the experiments, at least one of the two clones suffered strong competitive suppression during mixed infections. It was observed that the avirulent clone suffered from competition even when it infected mice before the virulent clone, whereas the virulent clone suffered from competition only when infecting mice after the avirulent clone. It was suggested that host immunity along with competition for resources played an important role in causing the suppression of one of the clones during mixed infections. However, the extent of the contribution of resource limitation and host immune response to competitive suppression could not be disentangled.

In a recent paper examining competition between malaria clones we found direct experimental evidence of immune-mediated competition [20]. This was the first evidence of such competition in any host-parasite system. Two genetically distinct clones of *P. chabaudi* (AS and AJ) were co-infected into mice. The AS clone is less virulent than the AJ clone, being associated with a lower peak parasitaemia, less RBC loss and less weight loss [21]. In order to determine if the immune response mediated competitive suppression, both immuno-competent and immuno-deficient (T-cell depleted) mice were infected. If competition was mediated by the immune response, then the expectation was that competitive suppression would be weaker in immuno-deficient mice than in immuno-competent mice. Compared to single clone infections, the presence of the AJ clone in mixed infections competitively suppressed the AS clone. Importantly, suppression was alleviated in immuno-deficient mice. The statistical analysis of the data, however, did not allow the determination of the nature, strength and precise timing of the suppression. Moreover, the data suggested that other competitive mechanisms must be important, although what those mechanisms were was impossible to determine.

Our aim in this paper is to re-examine this dataset using a dynamical model-based Bayesian inference approach in order to determine the nature of these competitive interactions, immune mediated or otherwise. Parameterised dynamical (process) mathematical models are fitted to the experimental data. Mechanism can then be inferred from the estimated parameters – i.e., a parameter for a mechanism (such as immune-mediated clearance rates of parasites) that is different across treatments suggests possible causes of competitive interactions [22]–[25]. This approach allows formal and quantitative testing and comparison of hypotheses for the effect of factors that cannot be easily measured empirically.

## Methods

### Experimental data

We briefly describe the experiment here. See [20] for a more detailed description.

Three different phenotypes of 12–14 week old, female BALB/c mice were used: (i) wildtype mice; (ii) female *nu/nu* mice (“nude mice”; Harlan UK); and (iii) nude mice reconstituted with T cells taken from wildtype mice. The mutation *nu* is a recessive mutation that blocks the development of the thymus and hence these mice have no mature T-cells which impairs their immune systems [26]. Both nude mice and nude mice reconstituted with T-cells are genetically different from wildtype mice. Only the nude and reconstituted mice were used in the analysis in [20] to allow for the comparison of genetically similar immuno-competent and immuno-deficient hosts; we present data for all three phenotypes here. The wildtype mice provide additional statistical power to discriminate between competing hypotheses about the cause of competitive suppression.

Mice of each phenotype (wildtype, nude, reconstituted) were inoculated intraperitoneally with 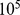 AS or 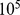 AJ or 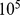 AS and 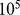 AJ parasitised RBCs (pRBCs); resulting in 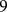 treatment groups. There were seven mice in the treatment groups with nude mice, and six mice in each of the treatment groups with reconstituted and wildtype mice. RBC and parasite densities were measured on days 0, 2, 4, and then daily until day 18 when the experiment was terminated. Measurements were taken at 08:00 hr before asexual merozoites have yet to replicate within pRBCs. RBC density was measured by flow cytometry, parasite density was measured by quantitative PCR. We have previously estimated the error in these measurements [25].

### The mathematical model and forms of competition

We extend the model of malaria parasite bloodstream asexual replication developed in [25] to mixed infection of two clones and further include RBC age-structure [22] and background loss of pRBCs. We provide a brief description of the model here; the mathematical details with supporting tables of variables, parameters and their priors are given in the Supplementary Materials.

In *P. chabaudi*, parasitised RBCs (pRBCs) rupture synchronously every 24 hours [27], releasing on average 6–8 parasites (merozoites) into the bloodstream [28]. These newly released merozoites infect further RBCs and the cycle repeats. The rupture of pRBCs (schizogony) occurs at approximately midnight [27], [29].

We use a discrete-time formulation to model the dynamics, where each time step corresponds to a single day. The start of day 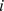 is defined as the point immediately following rupture of pRBCs, before any infection has occurred (i.e., the point at which merozoites are released into the bloodstream). The script for our model can be accessed at https://code.google.com/p/bayesian-model-based-inference/

We assume that the processes determining RBC and parasite densities occur on two non-overlapping timescales. The first corresponds to the short infection phase during which merozoites infect RBCs, which occurs within a few minutes following schizogony. The second and subsequent timescale (the remainder of the day) corresponds to the RBC turnover phase: the parasites replicate within pRBCs, and new unparasitised RBCs (uRBCs) migrate from the bone marrow and spleen into the bloodstream [24], [30], [31]. At the end of the RBC turnover phase, surviving pRBCs rupture and release new merozoites. In normal, homeostatic, conditions, migration of uRBCs exactly replenishes the natural loss of RBCs [32]. In anaemic conditions RBC production and migration (erythropoiesis) is up-regulated at a rate proportional to the difference between the normal RBC density and the actual density a few days in the past [22], [25], [33], [34]. As discussed below, one possible cause of competition is differential RBC-age preference between the two clones [35], [36]. We therefore extend the model to include age structure of RBCs as in [22], [23]. We distinguish between 1–2 day old immature RBCs (reticulocytes) and the older mature RBCs (normocytes) they develop into.

We model separate, time-dependent, adaptive immune responses against merozoites in the infection phase [37], [38] and pRBCs in the turnover phase as in [25]. We tried three different functional forms for the clearance rates: piecewise linear (as in [25]), exponential and sigmoidal. In addition, we include a constant, low-level background loss rate of free merozoites in the infection phase as in [22], [25], [36], and a constant background loss rate of pRBCs. We also include time-dependent bystander killing of uRBCs in the turnover phase [25], [39], [40]. The mathematical details are given in the Supplementary Material.

Biologically, competition between clones can be mediated by several processes as listed in Table 1. The main difference between the mouse phenotypes is their immuno-competence. Hence, we expect to see an effect of phenotypes in processes that involve host immune response. This allows us to identify all processes including host-immune response that may play a role in competitive suppression. Previous modelling studies of *P. chabaudi* [22], [36] have shown that clone-specific RBC age preferences can cause competitive suppression of a less virulent clone, when virulence is a function of the age range of RBCs a parasite can invade. Our first hypothesis H_1_, considers this possibility (Table 1). In our model age-preference is modelled as different merozoite infection rates of reticulocytes and normocytes; 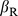 and 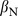 respectively. It turns out, however, that we cannot separately identify (estimate) these two rates; only their ratio 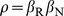, can be estimated (see Supplementary Material for details). Our second hypothesis H_2_, considers whether the number of merozoites that burst from pRBCs 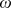, is different between clones. Evidence that burst sizes are significantly higher for the more virulent clones compared to avirulent ones have been observed previously [22], [23]. Our third hypothesis H_3_, considers the possibility that competition for resources within multiply parasitised RBCs may cause differential death rates 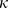, of the different clones. A previous *in vitro* study of *P. falciparum* has shown that diffusible molecules within RBCs can regulate the growth and gametocytogenesis of parasites [41]. Hence, multiple parasites within the same RBC may competitively interfere for these resources. Our fourth hypothesis H_4_, considers whether RBCs infected by the different clones have different constant background death rates 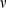. We do not have a specific process in mind that might cause such a difference, other than it not being caused by clone-specific adaptive immunity (which we consider in hypothesis H_7_). Our fifth hypothesis H_5_ considers competition caused by a combination of two processes: differential background loss rates of merozoites 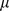, and differential merozoite infection rates of normocytes 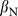. Mathematically we cannot separately estimate these two parameters; only their ratio 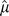, can be estimated (see Supplementary Material). The parameter 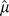 can be interpreted as the RBC density at which a single merozoite has a 50% chance of infecting a RBC (assuming no age preference, and in the absence of an adaptive immune response against merozoites). Hence, if one clone has a higher background merozoite loss rate or a lower merozoite infection rate of normocytes, this clone has a lower chance of infecting RBCs at a particular RBC density, and, therefore, is at a competitive disadvantage. In hypotheses H_6_ and H_7_ we consider clone-specific adaptive immunity against merozoites and pRBCs respectively.

**Table 1:** Possible causes of competition and associated clone-specific parameters.

Hypothesis	Cause of competitive suppression	Parameters that differ between clones
H_1_	Clone-specific RBC age preferences	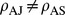
H_2_	Clone-specific burst sizes	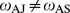
H_3_	Within-RBC interference competition	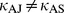
H_4_	Clone-specific background loss of pRBCs	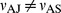
H_5_	Clone-specific ratio of background loss rate of merozoites to normocyte infection rates	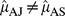
H_6_	Clone-specific adaptive responses against merozoites	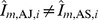
H_7_	Clone-specific adaptive responses against pRBCs	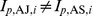

Competition is incorporated into the model via clone-specific parameters (Table 1). We would expect, after fitting the model to the data, for some of these parameters to exhibit different estimates between clones. We may then infer that competitive suppression is mediated by the processes whose parameters differ between clones. For example, the analysis by Råberg et al. [20] strongly suggested that competitive suppression was mediated by some aspect of immunity (hypotheses H_6_ and H_7_), so we might observe weaker adaptive immune clearance of the AJ clone compared to the AS clone.

We test the causes of competition as follows. The full model, described above and in more detail in the Supplementary Material, includes all possible causes of competition. That is, all parameters included in hypotheses H_1_ to H_7_ are allowed to be different between the two strains. This so called “all-cause” model is fit to the data. Each single-cause model is obtained by keeping the parameters clone-specific for the cause of interest and making parameters clone-non-specific for all other causes. Each single-cause model is fit to the data. If none of the single-cause models adequately predict the data, we would then examine dual-cause models, and so on. This was not necessary however. The all-cause model acts as a reference because it has the highest maximum likelihood. Any single-cause model that has a maximum likelihood similar to (but necessarily smaller than) the all-cause model fits the data as well as the all-cause model.

### Model fitting and parameter estimation

There is considerable variability in parasite and RBC dynamics of the mice both between and within the treatment groups. This suggests that there is variability in the underlying processes that govern the dynamics and thus in the parameters. Furthermore, the immune responses are significant sources of variability *in vivo* and RBC invasion rates may vary between-mice due to the multi-factorial nature of such processes which involve the interaction of many host and parasite proteins. We therefore make no assumption about which parameters are invariant across mice and estimate each parameter separately for each mouse.

In the experiment, measurements were taken at approximately 08:00 hrs, roughly 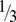 of the time between successive rupture events. We therefore fit the model predictions of RBC and parasite densities at this time. Total RBC density was measured by flow cytometry while the total parasite density was measured using quantitative PCR. We have previously shown that the measurement errors in RBC and log_10_-parasite densities are normally distributed with standard deviations 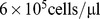 and 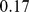 respectively [25]. Assuming independence in the errors, the likelihood of the model parameters, given the data for a particular mouse, is simply the product of the likelihoods of the parameters given each data point. We use an adaptive, population based Markov chain Monte Carlo method with power posteriors [24], [42]–[44] to sample the posteriors and compute marginal likelihoods (see below). The Markov chains had a burn-in of 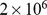 samples. Inferences are based on 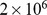 samples thinned to 3,000 samples. Five simulations were run to obtain means and standard errors of the marginal and maximum likelihoods.

### Model comparison

We use maximum and marginal likelihoods to compare our competing hypotheses about the causes of competitive suppression. Marginal likelihoods naturally penalise models that over-fit data with too many parameters. Marginal likelihoods are computed for each mouse [44]. Assuming mice are independent, the marginal likelihood over all mice is simply the product of their individual marginal likelihoods.

When comparing two hypotheses the ratio of their marginal likelihoods, their Bayes factor, is a convenient statistic. Bayes factors quantify how much more likely one hypothesis is over another given the observed data [45]. However, when comparing multiple hypotheses it is more convenient to compare the logs of their marginal likelihoods directly. A difference of 1 log would be strong evidence in favour of the more likely hypothesis, and a difference of 2 logs or more would be decisive evidence [45].

## Results

### Experimental data

The experimental data on nude and reconstituted mice are discussed in [20], [25]. We present the data here in a different format and present the previously unpublished data of infections in wildtype mice.

**Figure 1:**
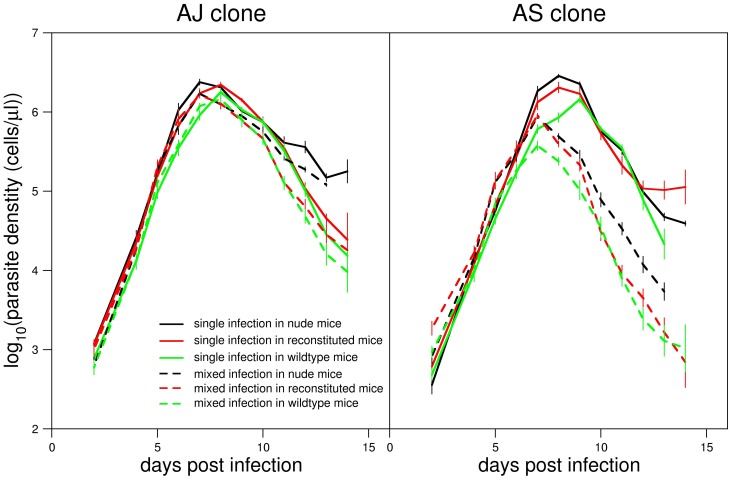
Data. Parasite densities of AJ and AS clones averaged across mice from single and mixed infections for three treatment groups (reconstituted, nude and wildtype). Error bars represent 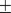
1 standard error. Data from [20].

The average parasite densities for the three mouse phenotypes for single (solid lines) and mixed (dashed lines) infections of the AJ (left panel) and AS (right panel) clones are shown in Figure 1. The results clearly demonstrate the strong competitive suppression of the AS clone in mixed infections (dashed lines) compared to single infections (solid lines) [20]. This is the case for all mouse phenotypes. The AJ clone, in comparison, does not exhibit any significant changes in parasite density during mixed infections when compared to single infection.

Figure 1 also shows that the strength of competitive suppression of the AS clone is stronger in immune-competent mice. This is seen by comparing the diverging densities of the AS clone in nude (dashed black line) and reconstituted mice (dashed red line). This result suggests that the AS clone undergoes immune mediated competition [20].

### Assessment of the all-cause competition model

The all-cause competition model was fit to the single and mixed infection data from [20]. The analysis of the fits to single infections has been reported elsewhere [25] so we only assess the fits to the mixed infections here.

We first tested the fits for the three functional forms of the adaptive immune responses. The sigmoidal response gave the best fits in terms of maximum and marginal likelihoods (see Table 2), the piecewise linear response gave slightly worse fits, and the exponential response gave significantly worse fits. For the rest of the paper we analyse the fits of the sigmoidal model. The results and conclusions from using the piecewise linear model are identical. We do not consider the exponential model any further.

**Table 2:** Assessment of functional forms of the adaptive immune responses.

Functional form	Maximum log-likelihood	Marginal log-likelihood
Sigmoidal	−3502±3^1^	−4275±2
Piecewise linear	−3513±3	−4289±2
Exponential	−3589±5	−4597±1

1 Mean
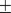
2 standard errors of five independent fits.

The standardised residuals of the all-cause model for each mouse phenotype are given in the Supplementary Material (Figs. S3, S4 and S5). The Q-Q plots of the all-cause model for each phenotype are given in the Supplementary Material (Figs. S6, S7, S8). The standardised residuals of an adequate model should be approximately normally distributed with mean 0 and standard deviation 1. The overlaid residuals and the Normal Q-Q plot of the fits suggest that the all-cause model is adequately fitting the data with some minor over and under estimation of the dynamics. We can therefore be confident that the all-cause model is adequately explaining the data and so we proceed to the single-cause models.

### Comparison of single-cause competition models

**Figure 2:**
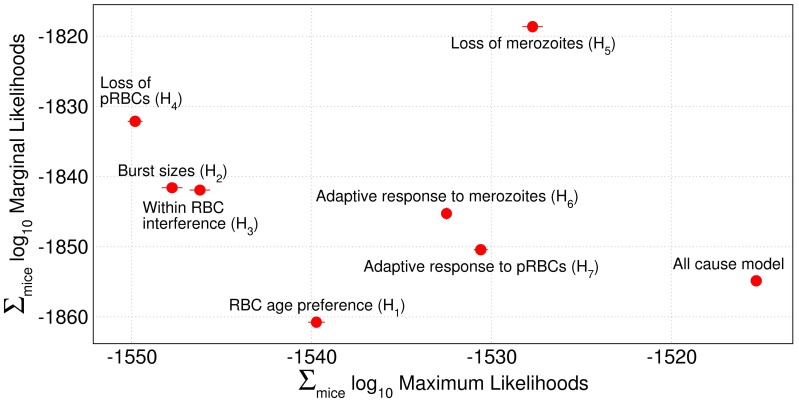
Statistical comparison of possible causes of competition. Marginal against maximum likelihoods on a 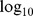 scale of the all-cause model and all single-cause models. See Table 1. As all mice are independent, the marginal and maximum likelihoods of a model are summed over all mice in all treatment groups. Competitive suppression of the AS clone by the AJ clone can be solely explained by differences in the parameter 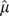 (Hypothesis H_5_). No other single cause of competition adequately predicts the data. Circles show mean, and error bars show 2 standard errors from 5 independent fits.

Figure 2 plots 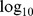-marginal likelihood against 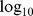-maximum likelihood of the models tested in Table 1. The all-cause model must have the highest maximum likelihood amongst all our models because it has the most degrees of freedom. We would expect, though, for it to have a low marginal likelihood due to over-fitting. The single-cause models may fall into one of two categories. i) A model may have a substantially poorer fit than the all-cause model causing it to have a substantially lower maximum likelihood. Its marginal likelihood may be lower or higher than the all-cause model. ii) A model may have almost as good a fit as the all-cause model causing it to have a similar maximum likelihood to it and a substantially higher marginal likelihood. Models falling into the latter category are considered minimal adequate models: they predict the data well with as few parameters as possible [46].

It is clear from Figure 2 that only one model falls into the minimal adequate category. The model with clone-specific differences in 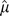 has a maximum likelihood slightly smaller than the all-cause model, meaning that it predicts the data almost as well. Its marginal likelihood is much higher because it has far fewer parameters. All other models can cause competition (results not shown). However, either their maximum likelihoods are at least an order of magnitude lower or their marginal likelihoods are significantly lower. Figures S9, S10, S11 in the Supplementary Material show marginal against maximum likelihoods for the three mouse phenotypes separately. In all, the model with clone specific differences in 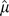 consistently has the highest marginal likelihood and similar maximum likelihoods to the all-cause model. We can thus conclude that clone-specific differences in 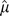 are sufficient to adequately explain the competitive suppression of the AS clone. All other parameters can be assumed to be the same between the two clones.

### Model fits of the minimal adequate model

**Figure 3:**
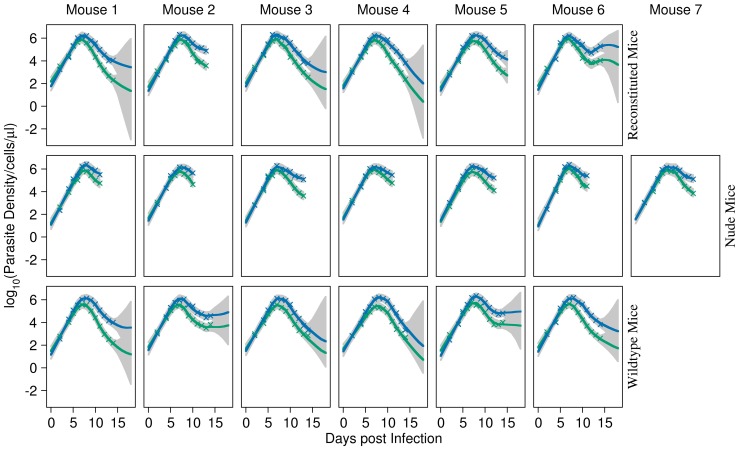
Model fits to parasite densities. Fits of the single-cause model (H_5_) with clone-specific 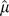 to AS (green) and AJ (blue) parasite densities in reconstituted (top panels), nude (middle panels) and wildtype (bottom panels) mice during mixed infections. Crosses are data. The solid lines give the median fits. Grey regions correspond to the 95% posterior intervals of model uncertainty. These plots show that the model fits the data quite well for each individual in all treatment groups.

**Figure 4:**
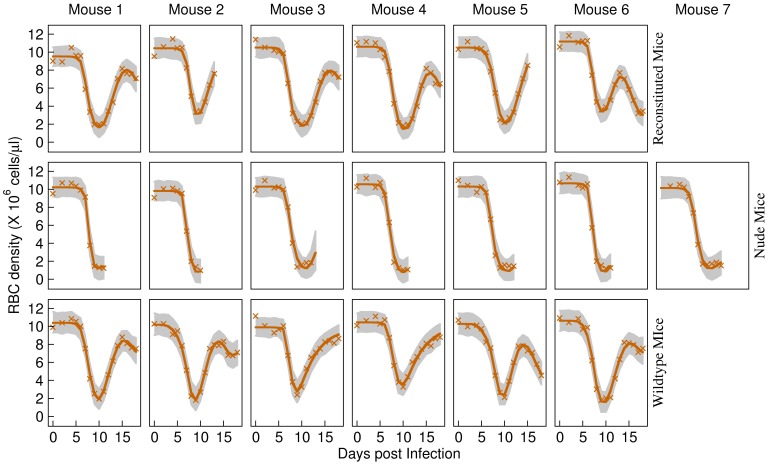
Model fits to RBC densities. Fits of the single-cause model (H_5_) with clone-specific 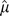 to RBC densities in reconstituted (top panels), nude (middle panels) and wildtype (bottom panels) mice during mixed infections. Crosses are data. The solid lines give the median fits. Grey regions correspond to the 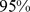 posterior intervals of model uncertainty. These plots show that the model fits the data quite well for each individual in all treatment groups.

The fits to individual mice data of the single-cause model with clone-specific 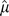 are shown in Figs. 3, 4. RBC density in reconstituted and wildtype mice recover after the first peak in parasite density, but then recrudesce around day 14 post infection. By comparison, RBC density does not recover in nude mice and they die.

### Statistical analysis of 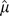 in the minimal adequate model

**Figure 5:**
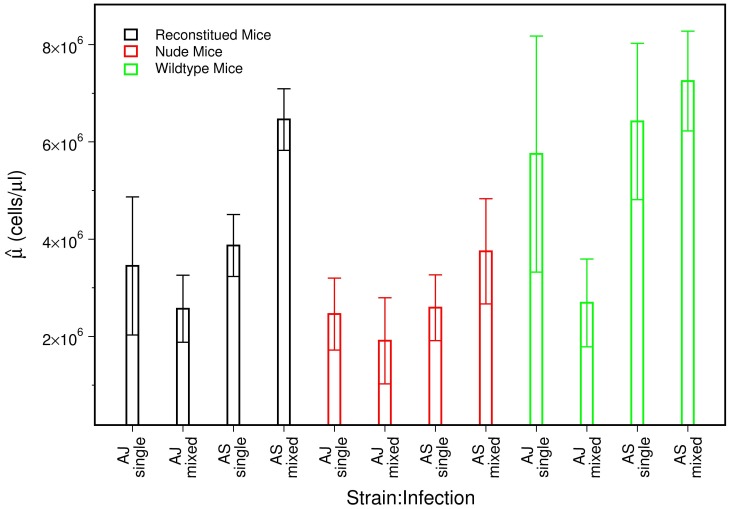
Estimates of 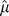, in reconstituted (6 mice), nude (7 mice) and wildtype (6 mice) in single and mixed infections. Bars represent the means of the means of the marginal posteriors. Error bars represent 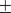
1 standard error.

Figure 5 shows the means (and their standard errors) of the posterior means of 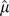 among mice within each treatment group. There are six features in Figure 5 that are pertinent for understanding how 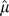 contributes to competitive suppression of the AS clone.

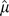
 is significantly higher in wildtype mice than in reconstituted mice (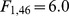, 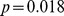) and significantly higher in reconstituted mice than in nude mice (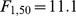, 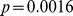).
2. In single clone infections there is no significant difference between 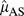 and 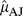 (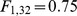, 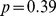).
3. In contrast to single infections, in mixed infections 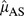 is significantly higher than 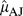 (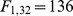, 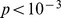).
4. In mixed infections, the difference between 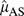 and 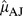 is significantly larger in reconstituted and wildtype mice than in nude mice (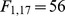, 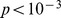). Between reconstituted and wildtype there was no significant difference in the difference between 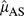 and 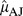 (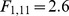, 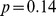).
5. In nude and reconstituted mice 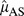 is significantly higher in mixed infections than in single infections (nude: 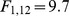, 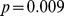, reconstituted: 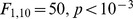). However, there is no difference in 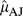 between single and mixed infections (nude: 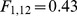, 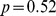, reconstituted: 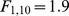, 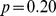).
6. In wildtype mice, the opposite is the case: 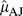 is significantly lower in mixed infections than in single infections (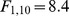, 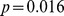), whereas there is no difference in 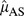 between single and mixed infections (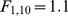, 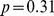).

We discuss the significance of these results next.

## Discussion

In mixed infections of virulent AJ and avirulent AS *P. chabaudi* clones, the AJ clone competitively suppresses the AS clone [20]. This competition is thought to be mediated partially by the immune response because in immune-deficient mice competitive suppression is alleviated [20]. The aim of this paper was to provide a quantitative assessment of the, possible, multiple factors that cause this competition. Drawing on hypotheses from experimental data and the mathematical modelling literature we built dynamical models and fitted them to the experimental data. The outputs were analysed using a Bayesian inference approach.

We tested seven possible mechanisms that could cause competitive suppression (Table 1). Our results suggest that just one model parameter 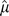, the ratio of background loss rate of free merozoites to their infection rate of normocytes, needs to be clone-specific in order to fully explain competition between the AS and AJ clones.

In fact, all of the mechanisms of competition we tested could explain competitive suppression (results not shown). However, these mechanisms did not predict the data as well as a clone-specific 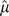 (see Figure 2). This does not imply that clone-specific differences in these other mechanisms do not exist. Other modelling work has suggested that clone-specific RBC age preference could cause competitive suppression [22], [6], [47], [48]. In these papers models were fitted to data from single-clone infections and the resulting estimated clone-specific, age-dependent infection rates used to *simulate* parasite and RBC dynamics in mixed infections. These simulations gave qualitatively similar dynamics to data from mixed infections thus suggesting that RBC age preference can cause competitive suppression. We went a step further in this study by fitting our model to the mixed infection data as well as the single infection data. This allowed us to quantitatively compare this mechanism with many others and demonstrate that, although it can explain competitive suppression, it does less well than clone differences in 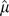.

Before we discuss the biological interpretation of 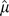 we first discuss the differences in its estimates across treatments (refer to Figure 5). In single infections, we found no difference in 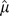 between clones (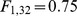, 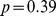). In mixed infections, however, 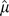 is significantly higher for AS than AJ (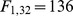, 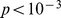). The difference between 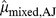 and 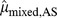 was significantly smaller in immune-compromised nude mice than in immune intact mice, both T-cell reconstituted and wildtype mice (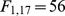, 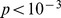). Therefore, we suggest that the reason why AJ competitively suppresses AS is because of clone-specific differences in 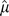, and the reason why competitive suppression is stronger in immune-intact mice is because the difference is larger in these mice.

In addition, in nude and reconstituted mice we found that 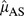 significantly increased between single and mixed infections (nude: 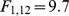, 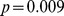, reconstituted: 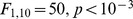). Whereas 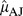 did not significantly change between single and mixed infections (nude: 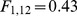, 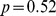, reconstituted: 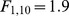, 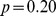). The opposite was the case in wildtype mice: 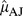 significantly decreased between single and mixed infections (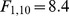, 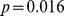) whereas 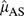 did not significantly change (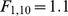, 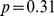). We can offer no explanation for this qualitative difference between mice phenotypes, other than to note that nude mice and nude mice reconstituted with T-cells are genetically different from wildtype mice.

Our definition of 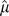 is the ratio of background loss rate of merozoites 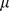, to the infection rate of normocytes 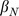. Thus it determines how many merozoites successfully invade RBCs; the larger its value the fewer merozoites which are successful. Moreover, because 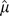 is assumed constant throughout the infection, its effect on parasite and RBC dynamics is felt from the first day of infection. Its effect on parasite dynamics is three fold. 1) It slows growth during the exponential growth phase (compare green (AS) and blue (AJ) lines in Figure 3). 2) This in turn determines the peak parasite density. This is because the timing and strength of the adaptive immune response is the same for both clones and adaptive immunity is the most important driver for halting and reversing parasite growth. If growth is slower (due to a larger 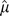) then peak parasitaemia will be lower. 3) It speeds up the loss of parasites after the peak. All of the differences in the dynamics between the two clones in Figure 3 are due to clone-specific differences in 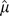, all other parameters, apart from initial parasite density, are non-specific.

The parameter 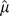 can be mathematically interpreted as the RBC density at which a single merozoite has a 50% chance of infecting a RBC (assuming no age preference, and in the absence of an adaptive immune response against merozoites). But how do we interpret it biologically? We initially defined it to be the ratio of background loss rate of merozoites 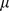, to the infection rate of normocytes 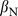. The definition of 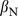 is straightforward and has been used in one form or another in all published mathematical models of malaria parasite invasion of RBCs; it parameterises the rate at which merozoites infect normocytes in the absence of an immune response. Our definition of 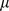 is based on the models of Mideo et al. [22] and Antia et al. [36]. These two papers base the value of 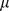 on *in vivo* measurements of the loss of invasive ability of free merozoites [49]. These two papers fix the value of 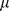 and therefore do not estimate its value, which we do here. Thus 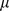 has always been defined as a property of the parasite and not as a property of the interaction between host and parasite.

Our finding that 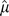 changes between single and mixed infections does not fit with the above definitions of 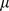 and 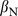. We can think of no valid reason why 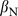 should change between single and mixed infections. It is unlikely that different parasite clones could interfere with each others ability to find, attach and infect RBCs, especially when they are at very low densities early in the infection. It is possible that antibody against one clone could block the invasion of RBCs by another clone thus changing 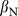. However, we observe competitive suppression before an antibody response is activated as well as in T-cell deficient nude mice. Thus it seems unlikely that 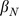 is changing between single and mixed infections.

This leads us to suggest that our definition of 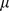 is at fault. It is likely that 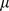 represents a combination of factors. We argue that one of these factors could be the innate immune response's clearance of free merozoites, and it is this factor that changes during mixed infections. First, 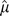 is weakest in nude mice and strongest in wildtype mice (Figure 5, nude vs. reconstituted: 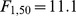, 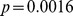, wildtype vs. reconstituted: 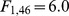, 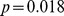) which suggests that 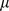 represents the ability of the immune response to clear parasites. Second, the relative difference between 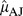 and 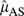 is larger in immune-competent mice than in immune-compromised mice (Figure 5, 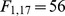, 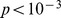) again suggesting that 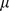 is determined by the immune response. Finally, *in vivo* experiments show that parasite growth rate in the exponential phase increases at low parasite dose and saturates at high parasite dose [50]. It was argued that this is because the innate response is limited in its ability to control large numbers of parasites [50]. Thus there is precedent for the argument that the strength of the innate response controls the growth in the exponential phase.

Although clone-specific differences in 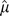 give the most probable fit to the data (Figure 2), we cannot rule out other clone-specific differences. In particular clone-specific adaptive immune clearance of merozoites and pRBCs. The models of these two hypotheses have an additional three parameters compared to the model of clone-specific 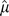. This explains their significantly lower marginal likelihoods. But even with more parameters they still do not fit the data quite as well as clone-specific 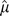 (Figure 2). This is for the following reason. As the mice have not experienced malaria parasites before, the adaptive immune clearance rate of parasites must be negliglible (we assume 0) on the day of inoculation. The clearance rate must grow over the course of infection leading to the rapid decline of parasite numbers about a week post infection. Therefore the effect of the adaptive response on parasite dynamics is negligible in the first few days post infection. Therefore a model of clone-specific differences in adaptive immune clearance cannot explain the differences in the growth rates of the clones seen in mixed infections. These differences in growth rates are small (Figure 1), hence the similarity in the maximum likelihoods between the models with clone-specific adaptive responses and clone-specific 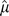.

Our results leave us with two unanswered questions: Why should the clearance rate of parasites by the innate immune response change between single and mixed infections? And why is the change in clearance rates positive for the AS clone in nude and reconstituted mice and negative for the AJ clone in wildtype mice (Figure 5)? We believe that the most likely answer to these two questions lies in the strength of cross reactive innate responses. The strength of the innate immune response to the parasite is determined by the density of parasites. Naturally the innate response to the AS parasite is higher in mixed than single infections. However, since AJ is the virulent clone, the addition of AS parasite in mixed infections has negligible effect on the total parasite density. Therefore, there is no extra stimulation of the density dependent response as a result of mixed infection. On the other hand, one could imagine that the innate response is dependent not only on density but also on the diversity of the infection, such that, more diverse infections are harder for the immune system to control. This could explain why the innate response against the AJ parasite in wildtype mice generally decreases in mixed infections when compared to single infections. One other possibility could be the interaction between innate responses triggered by schizogony of one clone adversely affecting the other due to the delay in schizogony of the affected clone. We are examining this idea with other data sets [27].

In conclusion, our dynamical model-based inference approach can be used to compare multiple hypotheses about biological processes underlying infection dynamics data. Using this approach we have shown that competitive suppression of an avirulent clone of *P. chabaudi* is most likely mediated through innate clearance of merozoites acting throughout an acute infection.

## Supporting Information

Figure S1 **Schematic of model showing the 24 hr cycle.** The model includes the erythropoiesis cycle where new uninfected reticulocytes are produced that mature into normocytes, and the erythrocytic phase of the parasites which includes the infection phase, RBC turn over phase and schizogony.
(TIF)

Figure S2 **Assessment of convergence of Markov chains.** Gelman-Rubin statistics for each parameter sorted by mouse (top panel) and by parameter (bottom panel). A statistic below 1.1 suggests excellent convergence of the Markov chains [51], [52].
(TIF)

Figure S3 **Standardised residuals of reconstituted mice.** Assessment of the all-cause model fits to the data by standardised residuals for reconstituted mice; AS parasite density (top panel); AJ parasite density (middle panel); RBC density (bottom panel). Each cross represents the standardised residual of a time point for an individual mouse. The solid red line joins the means of the standardised residuals at each time point. The dashed lines represent the 95% interval for the expected mean for the same number of residuals as the data (see [25] for details). The model systematically overestimates the data when the red line lies below the 95% interval, and underestimates the data when it lies above this interval. The y-axis is scaled in units of standard deviations.
(TIF)

Figure S4 **Standardised residuals of nude mice.** Assessment of the all-cause model fits to the data by standardised residuals for nude mice. See caption in Figure 8 for details.
(TIF)

Figure S5 **Standardised residuals of wildtype mice.** Assessment of the all-cause model fits to the data by standardised residuals for wildtype mice. See caption in Figure 8 for details.
(TIF)

Figure S6 **Q-Q plots for reconstituted mice.** The standardised residuals are approximately normally distributed suggesting adequate fits to the data. AS parasite density quantiles (top panel); AJ parasite density quantiles (middle panel); RBC density quantiles (bottom panel).
(TIF)

Figure S7 **Q-Q plots for nude mice.** The standardised residuals are approximately normally distributed suggesting adequate fits to the data. AS parasite density quantiles (top panel); AJ parasite density quantiles (middle panel); RBC density quantiles (bottom panel).
(TIF)

Figure S8 **Q-Q plots for wildtype mice.** The standardised residuals are approximately normally distributed suggesting adequate fits to the data. AS parasite density quantiles (top panel); AJ parasite density quantiles (middle panel); RBC density quantiles (bottom panel).
(TIF)

Figure S9**Statistical comparison of possible causes of competition for reconstituted mice.** Marginal against maximum likelihoods on a 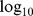 scale of the all-cause model and all single-cause models. See Table 1. As all mice are independent, the marginal and maximum likelihoods of a model are summed over all mice in all treatment groups. Competitive suppression of the AS clone by the AJ clone can be solely explained by differences in the parameter 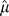 (Hypothesis H_5_).(TIF)

Figure S10 **Statistical comparison of possible causes of competition for nude mice.** See Figure S9 for details.
(TIF)

Figure S11 **Statistical comparison of possible causes of competition for wildtype mice.** See Figure S9 for details.
(TIF)

Table S1**Model variables.** Dependence on day 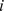 is dropped for clarity.(PDF)

Table S2 **Model parameters.** ^1^Dependence on AS and AJ removed for brevity. ^2^ N_T_ is a Normal distribution truncated at 0.
(PDF)

Text S1 **Models and methods.** A detail description of the various models in the paper and the methods used to compare them.
(PDF)
