# Supplementary figures and images for: Immune-Mediated Competition in Rodent Malaria Is Most Likely Caused by Induced Changes in Innate Immune Clearance of Merozoites
Source: PLoS Comput Biol. 2014 Jan 23;10(1):e1003416. doi: 10.1371/journal.pcbi.1003416 (PMC3900382; doi:10.1371/journal.pcbi.1003416)

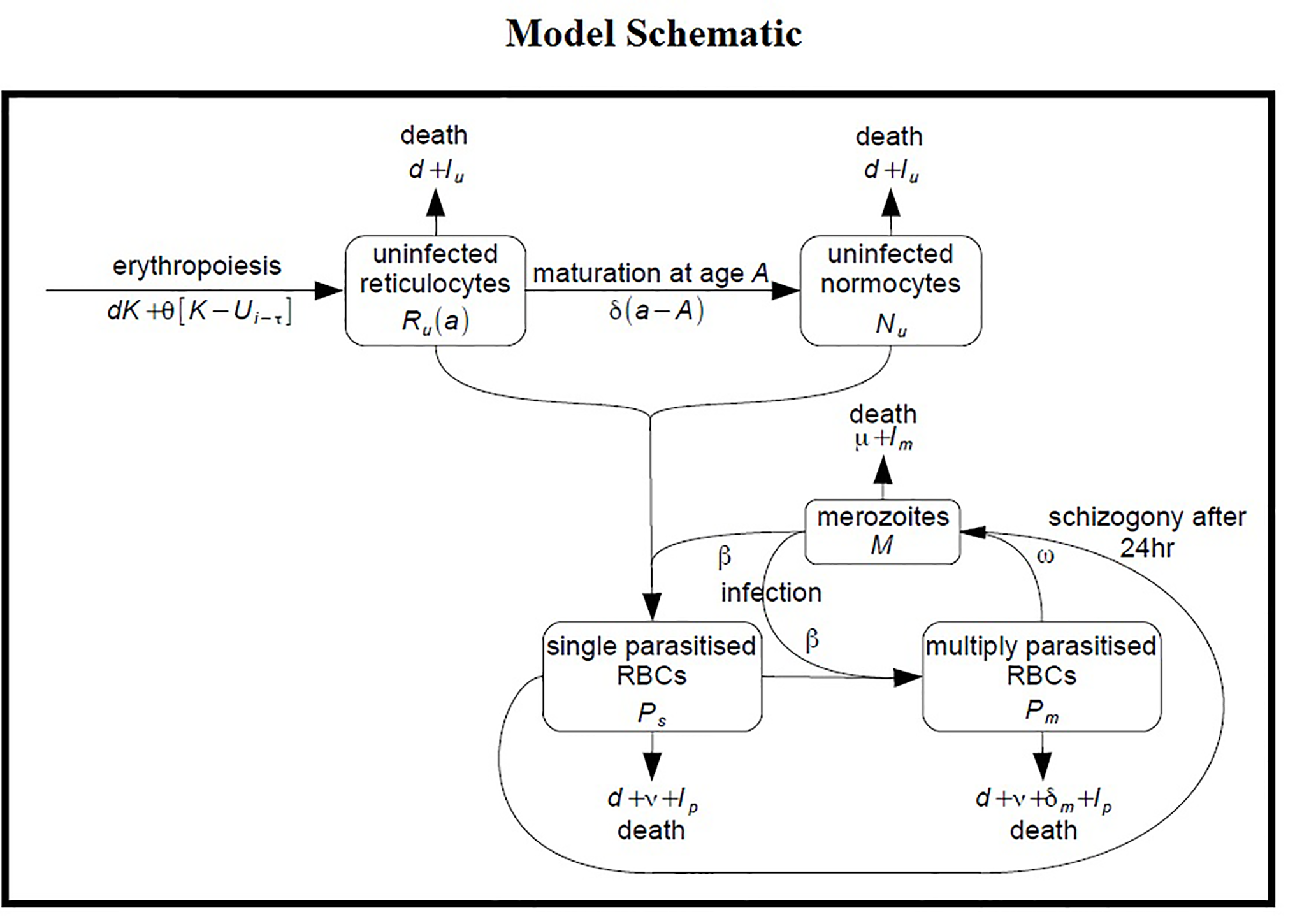

Supplement: Figure S1 — Schematic of model showing the 24 hr cycle. The model includes the erythropoiesis cycle where new uninfected reticulocytes are produced that mature into normocytes, and the erythrocytic phase of the parasites which includes the infection phase, RBC turn over phase and schizogony. (TIF) [file pcbi.1003416.s001.tif]

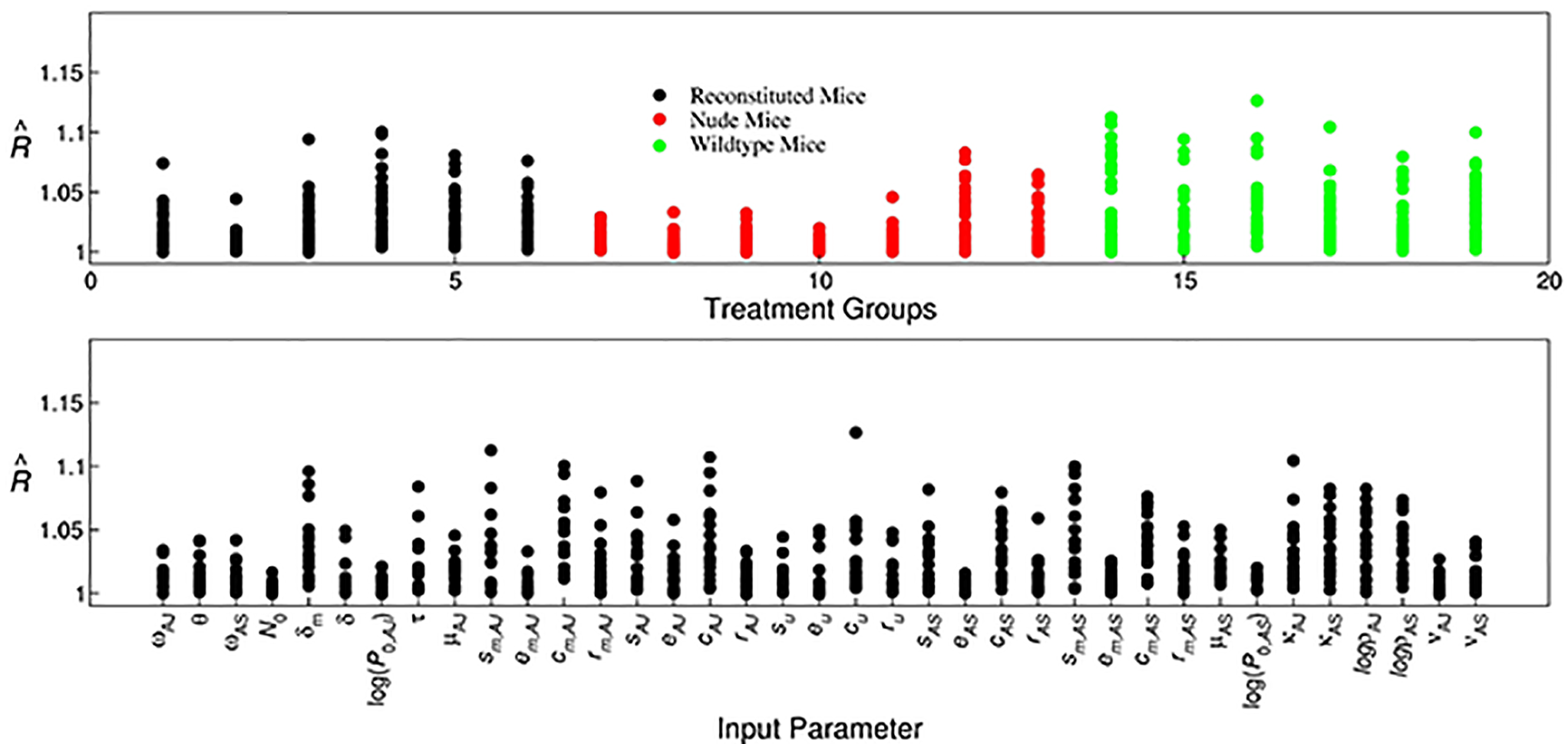

Supplement: Figure S2 — Assessment of convergence of Markov chains. Gelman-Rubin statistics for each parameter sorted by mouse (top panel) and by parameter (bottom panel). A statistic below 1.1 suggests excellent convergence of the Markov chains [51], [52]. (TIF) [file pcbi.1003416.s002.tif]

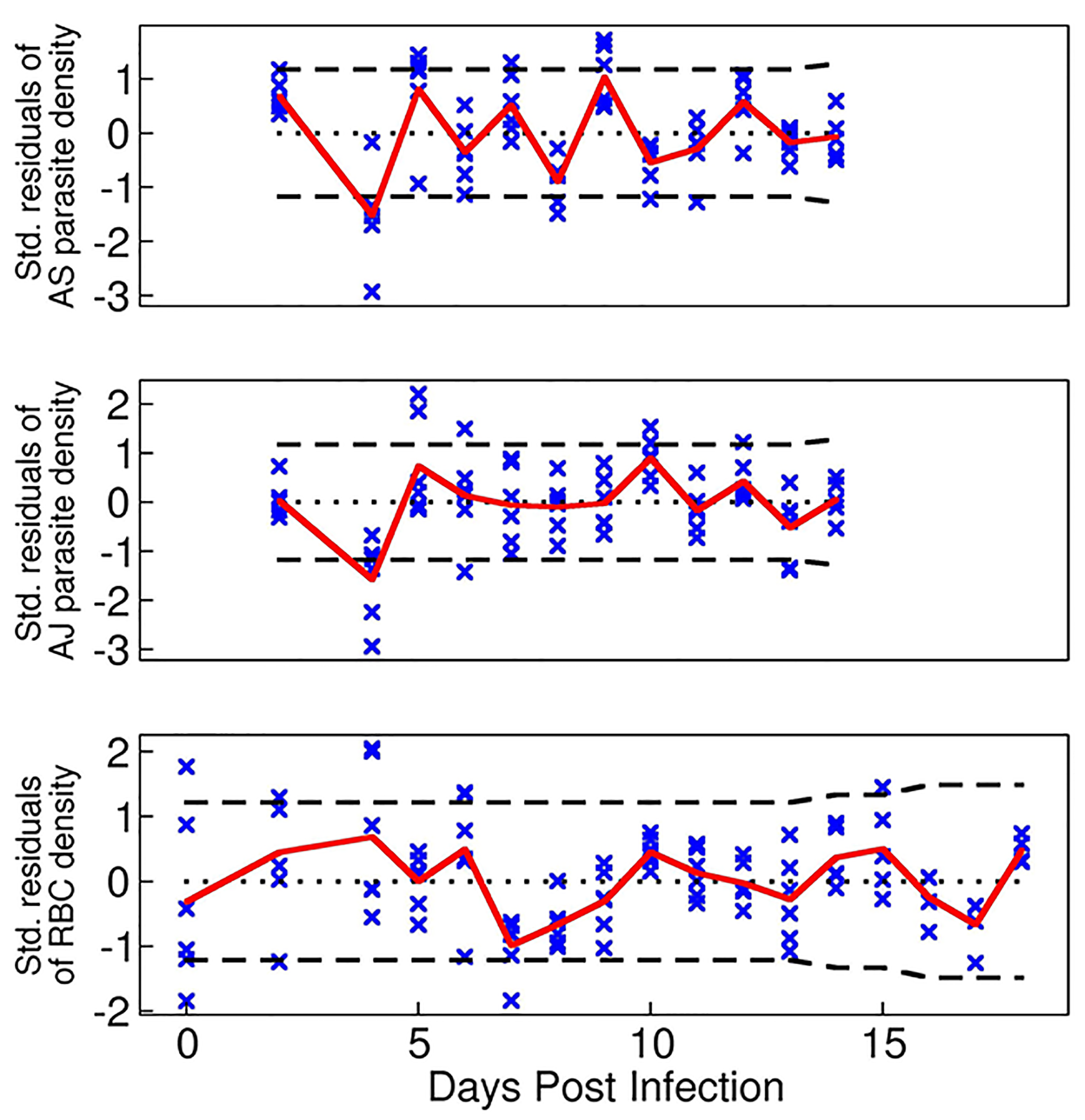

Supplement: Figure S3 — Standardised residuals of reconstituted mice. Assessment of the all-cause model fits to the data by standardised residuals for reconstituted mice; AS parasite density (top panel); AJ parasite density (middle panel); RBC density (bottom panel). Each cross represents the standardised residual of a time point for an individual mouse. The solid red line joins the means of the standardised residuals at each time point. The dashed lines represent the 95% interval for the expected mean for the same number of residuals as the data (see [25] for details). The model systematically overestimates the data when the red line lies below the 95% interval, and underestimates the data when it lies above this interval. The y-axis is scaled in units of standard deviations. (TIF) [file pcbi.1003416.s003.tif]

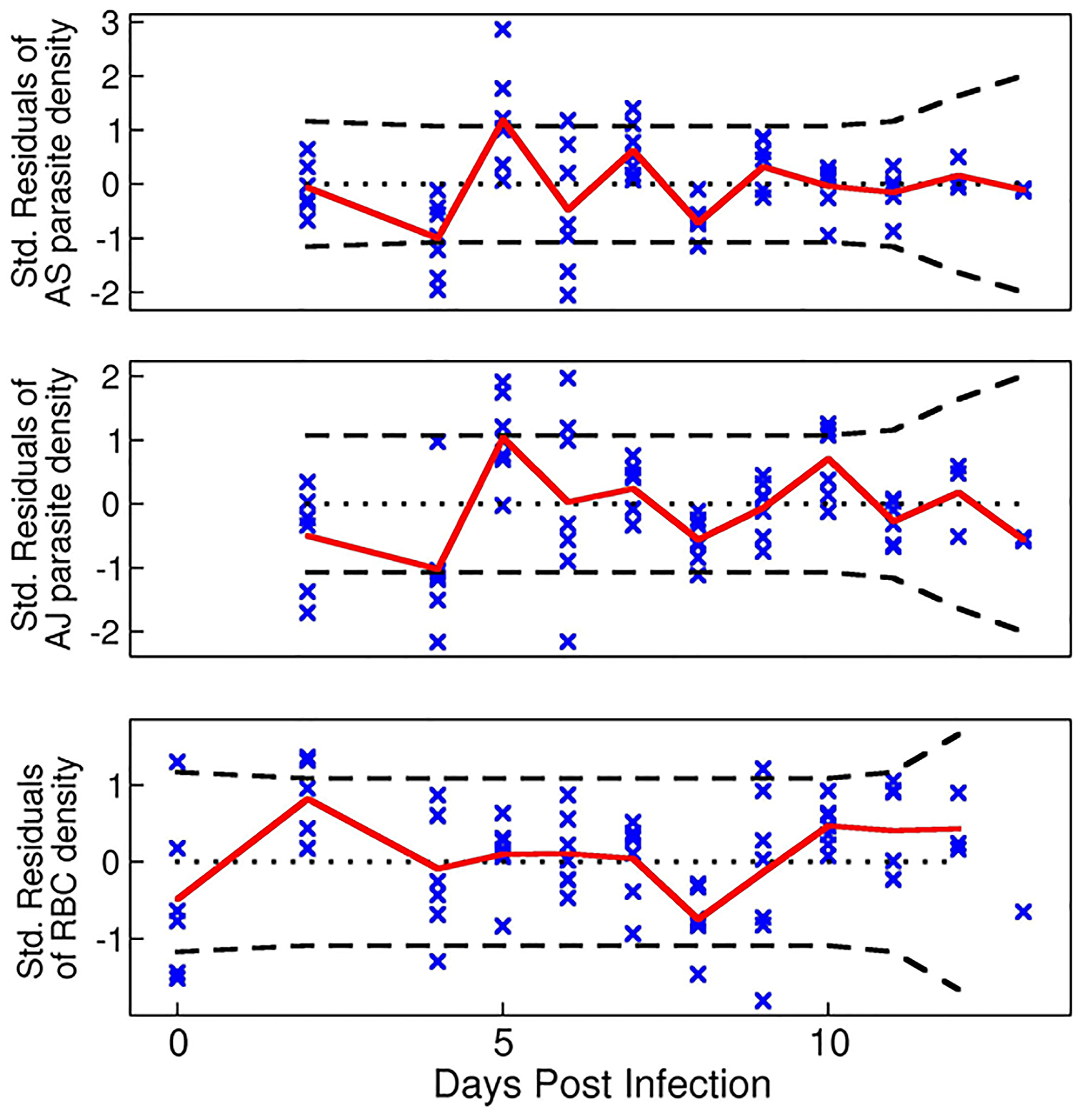

Supplement: Figure S4 — Standardised residuals of nude mice. Assessment of the all-cause model fits to the data by standardised residuals for nude mice. See caption in Figure 8 for details. (TIF) [file pcbi.1003416.s004.tif]

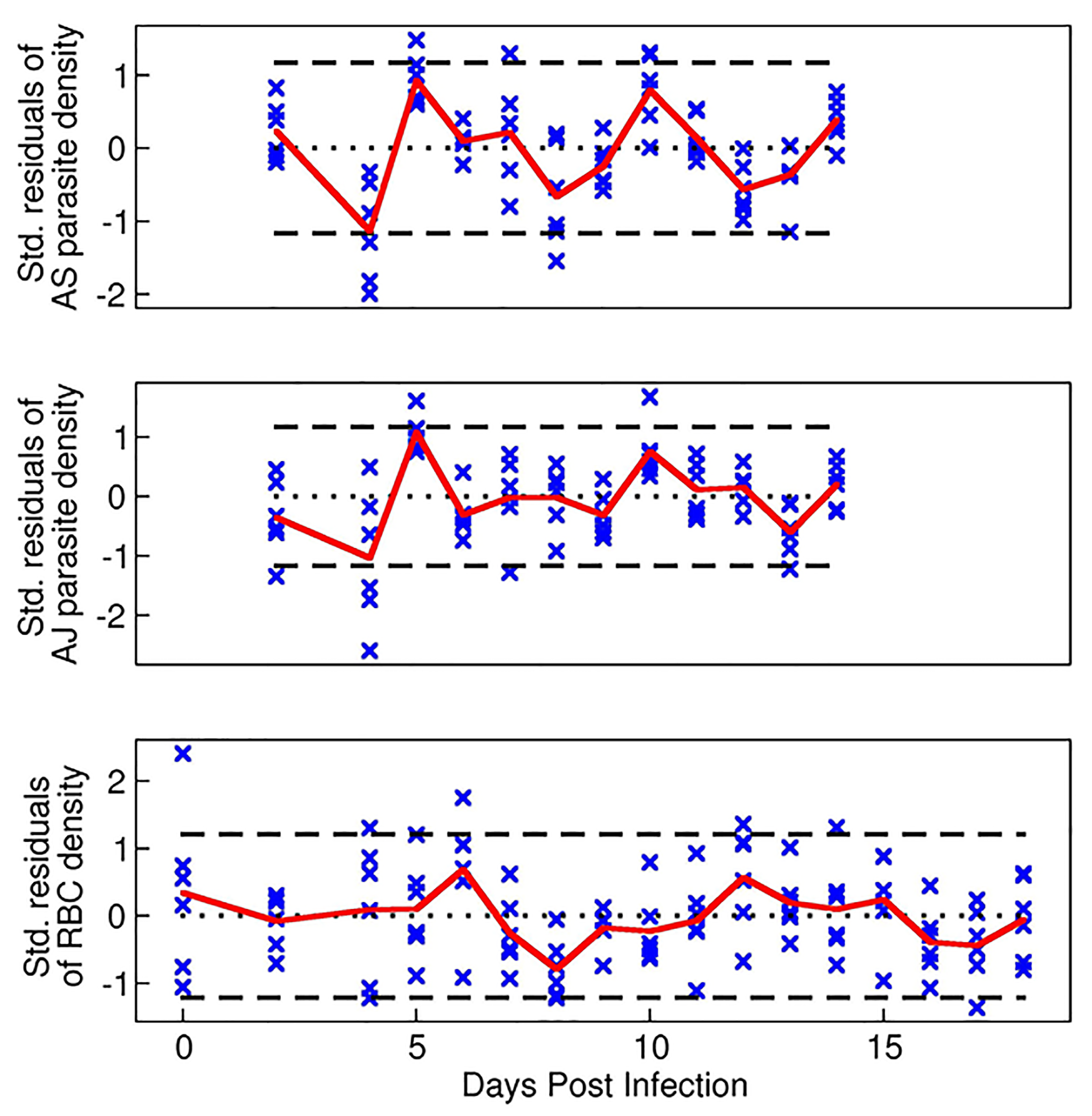

Supplement: Figure S5 — Standardised residuals of wildtype mice. Assessment of the all-cause model fits to the data by standardised residuals for wildtype mice. See caption in Figure 8 for details. (TIF) [file pcbi.1003416.s005.tif]

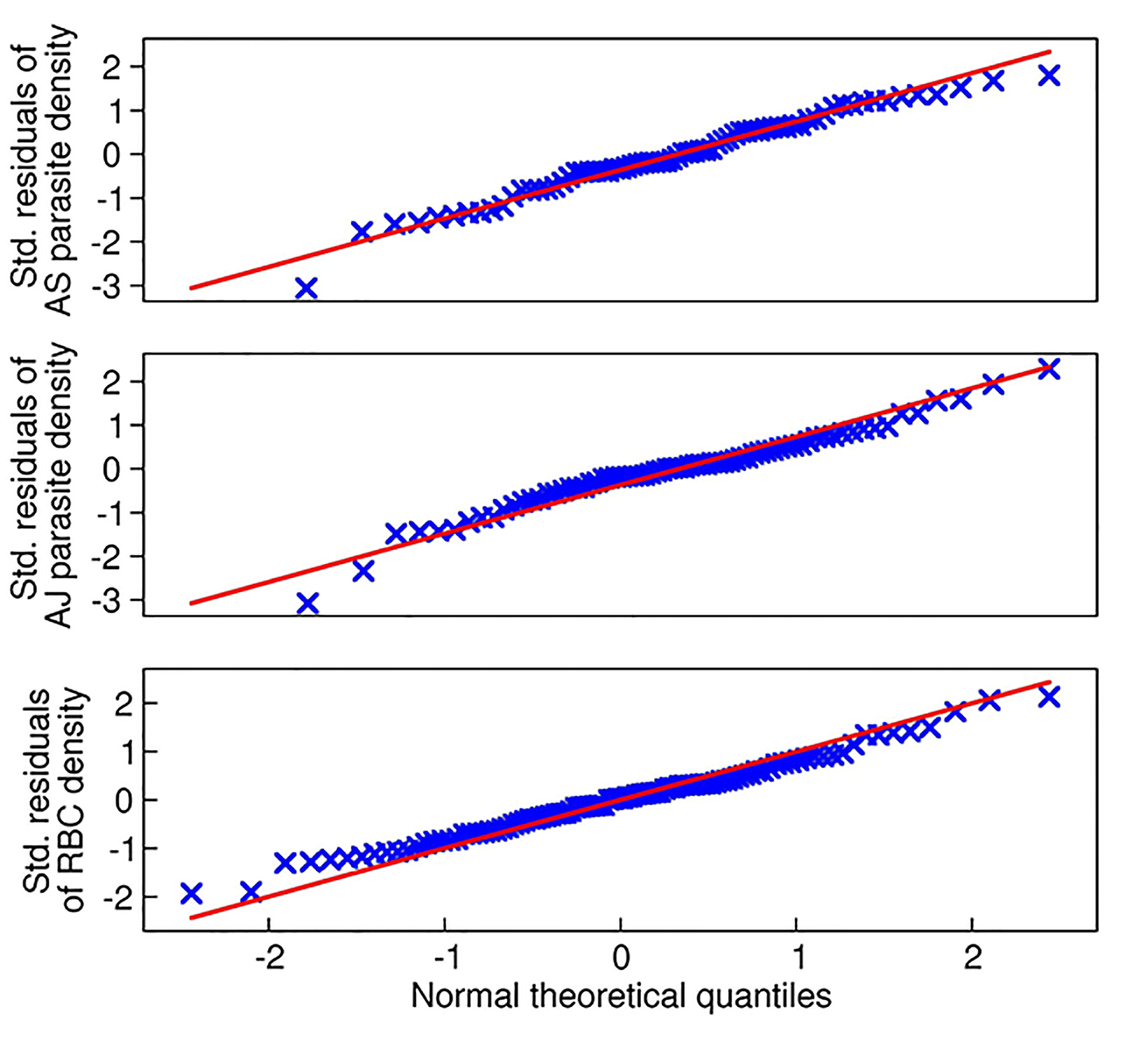

Supplement: Figure S6 — Q-Q plots for reconstituted mice. The standardised residuals are approximately normally distributed suggesting adequate fits to the data. AS parasite density quantiles (top panel); AJ parasite density quantiles (middle panel); RBC density quantiles (bottom panel). (TIF) [file pcbi.1003416.s006.tif]

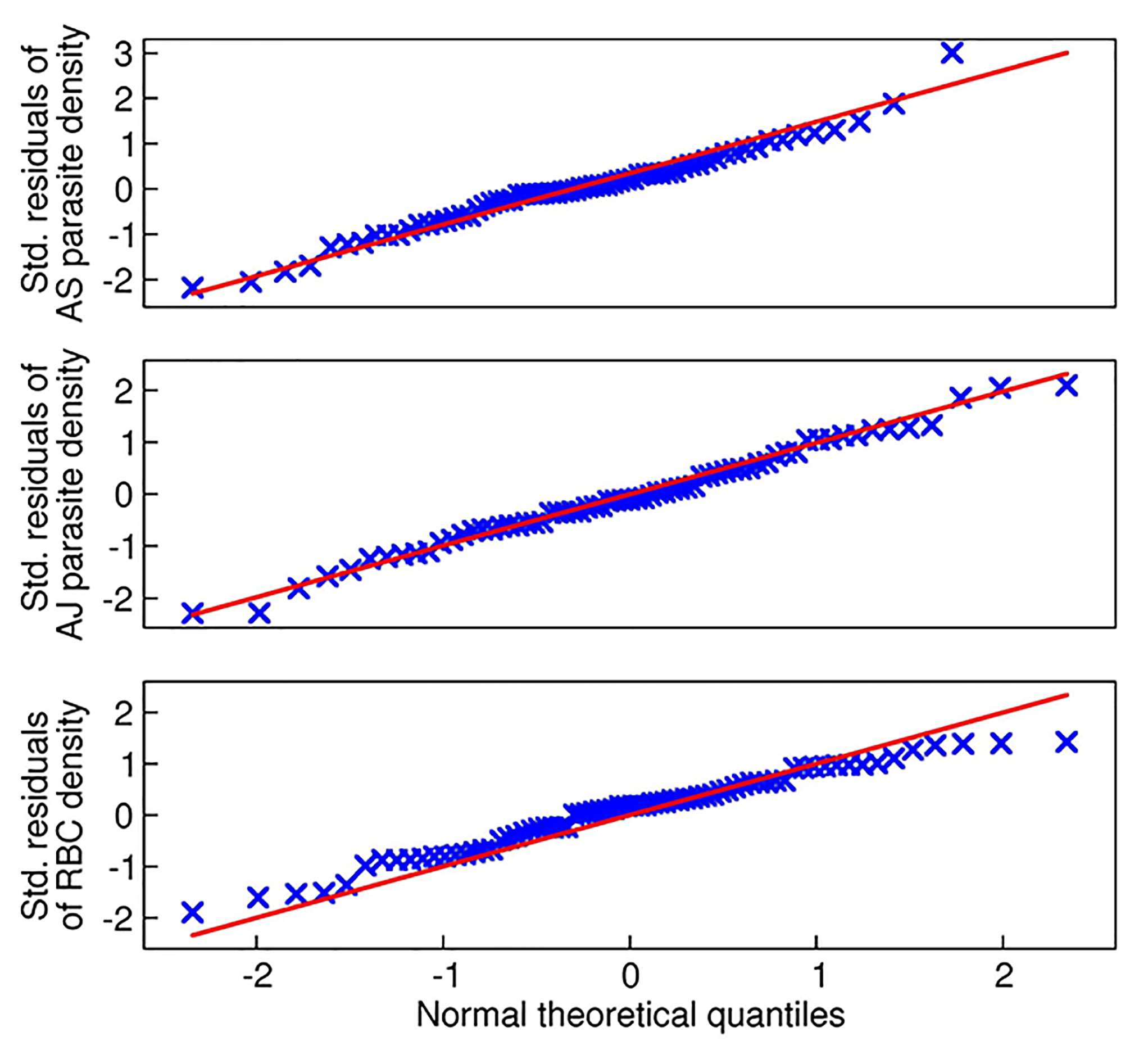

Supplement: Figure S7 — Q-Q plots for nude mice. The standardised residuals are approximately normally distributed suggesting adequate fits to the data. AS parasite density quantiles (top panel); AJ parasite density quantiles (middle panel); RBC density quantiles (bottom panel). (TIF) [file pcbi.1003416.s007.tif]

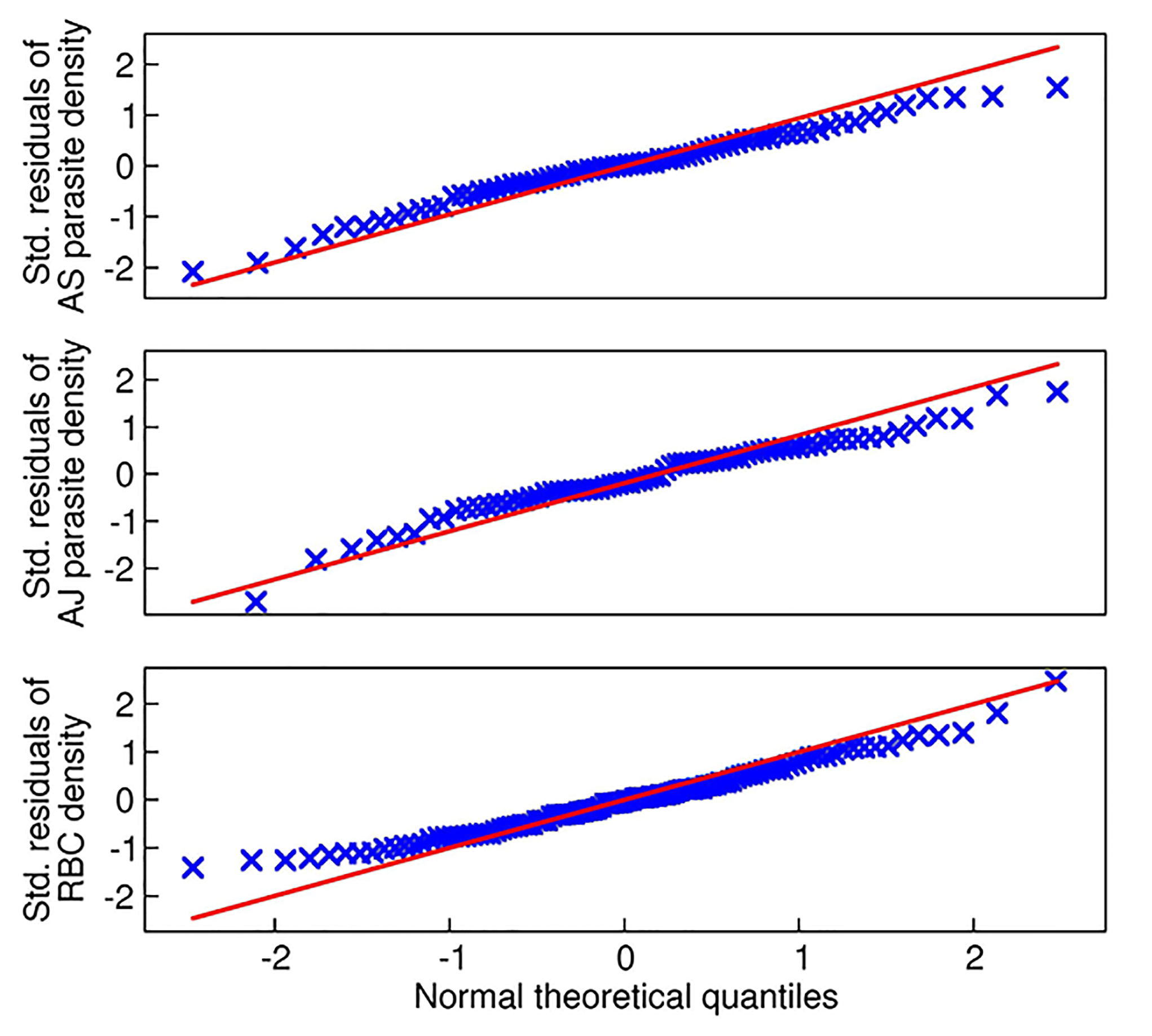

Supplement: Figure S8 — Q-Q plots for wildtype mice. The standardised residuals are approximately normally distributed suggesting adequate fits to the data. AS parasite density quantiles (top panel); AJ parasite density quantiles (middle panel); RBC density quantiles (bottom panel). (TIF) [file pcbi.1003416.s008.tif]

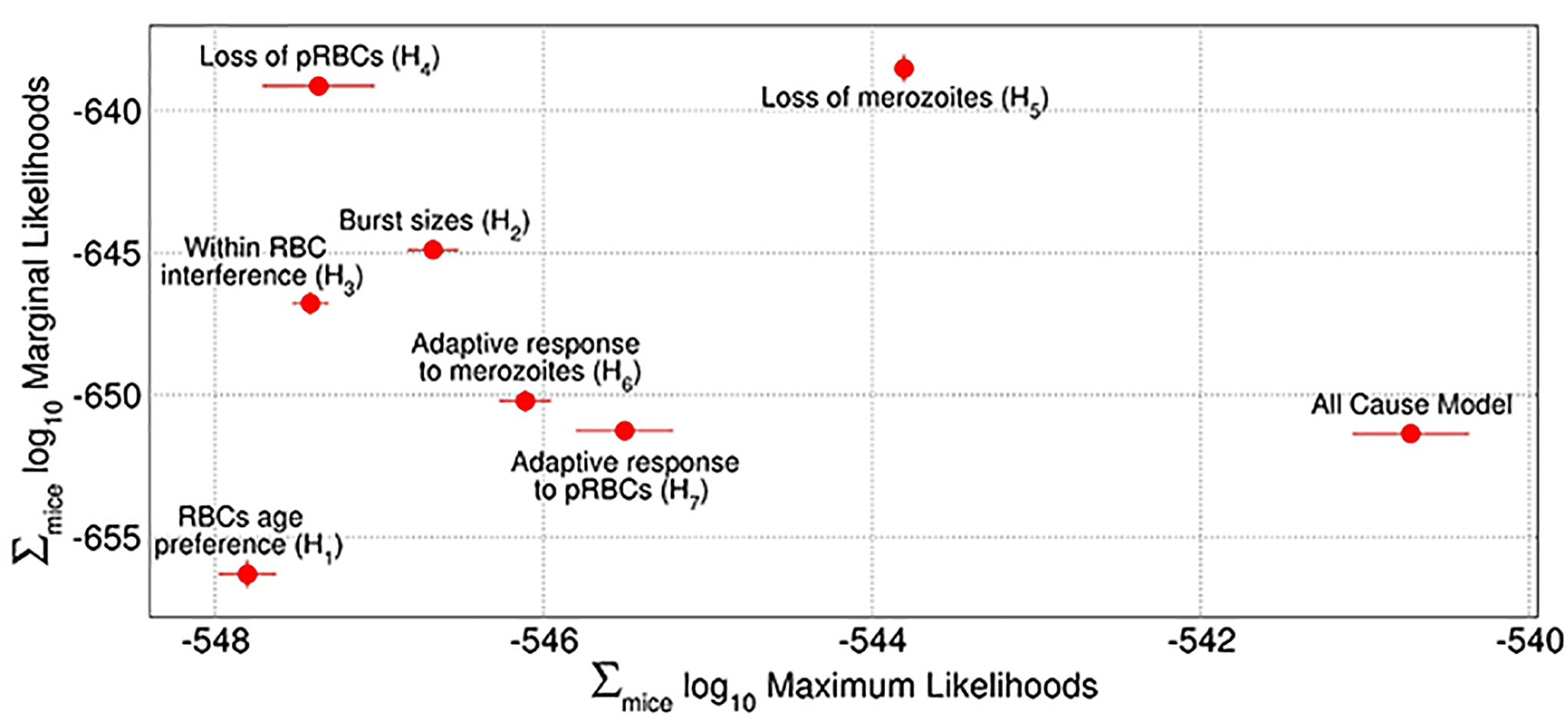

Supplement: Figure S9 — Statistical comparison of possible causes of competition for reconstituted mice. Marginal against maximum likelihoods on a scale of the all-cause model and all single-cause models. See Table 1. As all mice are independent, the marginal and maximum likelihoods of a model are summed over all mice in all treatment groups. Competitive suppression of the AS clone by the AJ clone can be solely explained by differences in the parameter (Hypothesis H5). (TIF) [file pcbi.1003416.s009.tif]

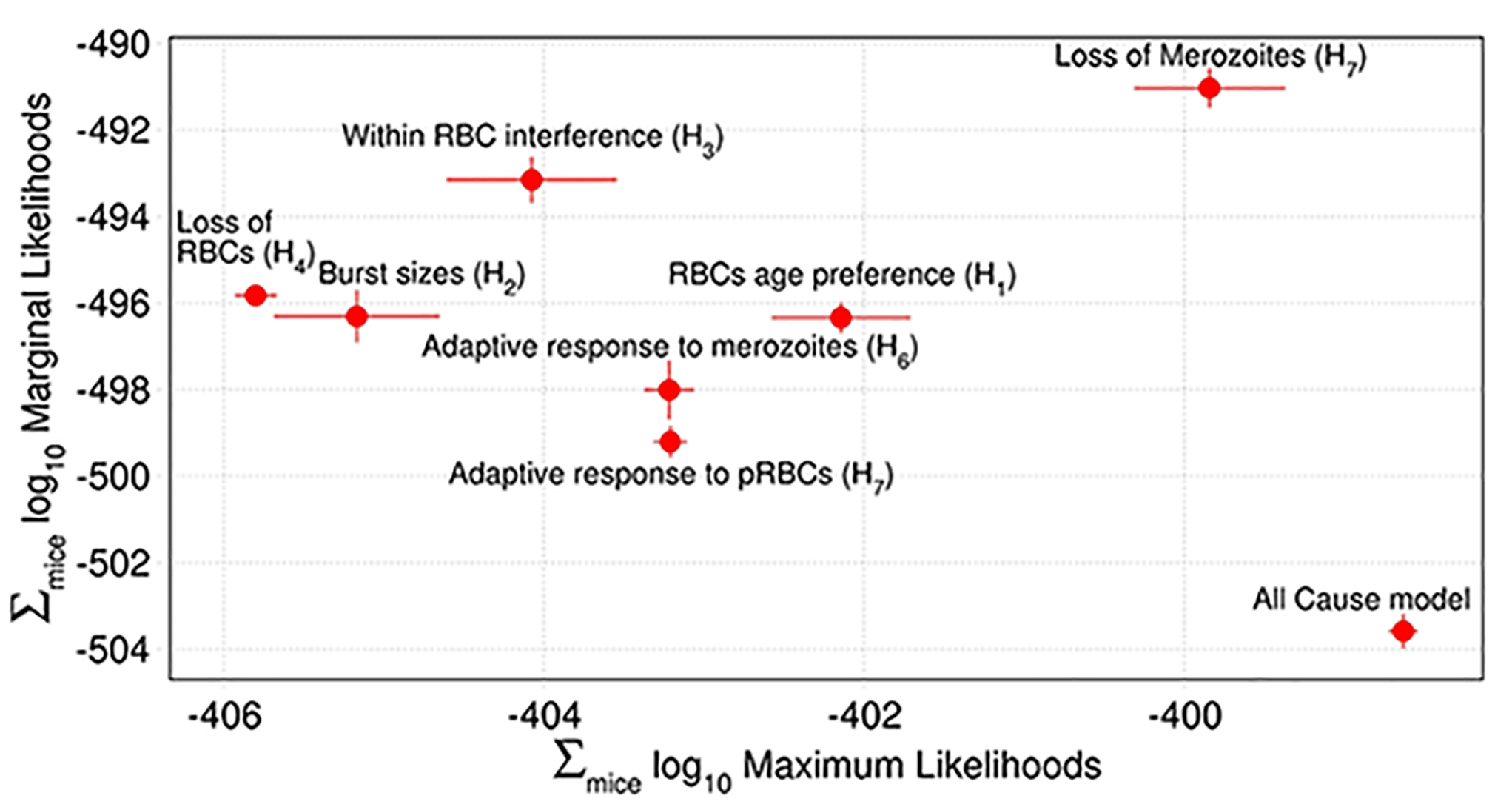

Supplement: Figure S10 — Statistical comparison of possible causes of competition for nude mice. See Figure S9 for details. (TIF) [file pcbi.1003416.s010.tif]

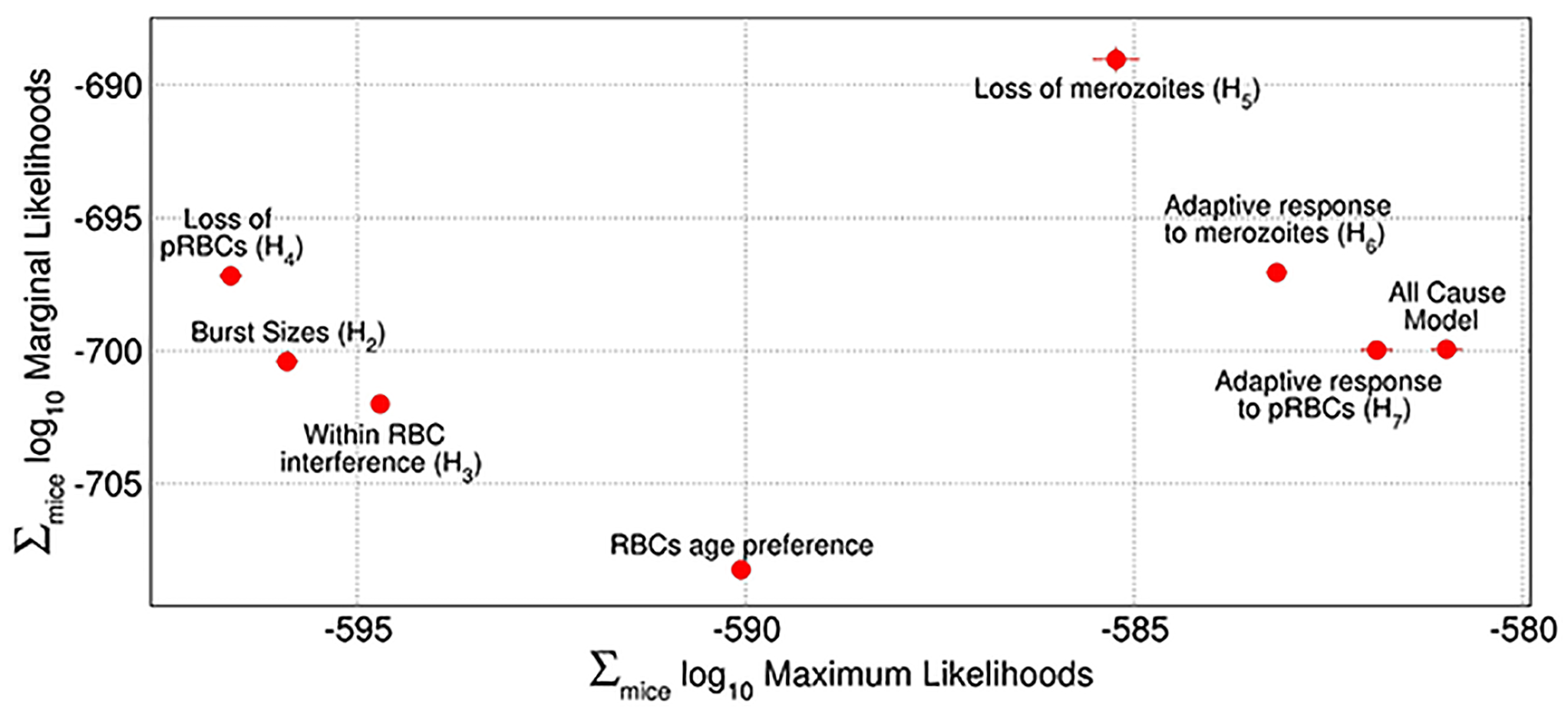

Supplement: Figure S11 — Statistical comparison of possible causes of competition for wildtype mice. See Figure S9 for details. (TIF) [file pcbi.1003416.s011.tif]
