# Supplementary material for: Immune-Mediated Competition in Rodent Malaria Is Most Likely Caused by Induced Changes in Innate Immune Clearance of Merozoites
Source: PLoS Comput Biol. 2014 Jan 23;10(1):e1003416. doi: 10.1371/journal.pcbi.1003416 (PMC3900382; doi:10.1371/journal.pcbi.1003416)
Supplement: Table S1 — Model variables. Dependence on day is dropped for clarity. (PDF) [file pcbi.1003416.s012.pdf]

Variable	Description	Units
**Independent variables**		
i	day post infection	
$t \in [0, 1]$	time	day
$a \in [0, A]$	reticulocyte age	day
**Infection phase variables**		
M_{AS}	density of free AS merozoites	parasites/μl
M_{AJ}	density of free AJ merozoites	parasites/μl
$r(a)$	density of unparasitised reticulocytes of age a	cells/μl/day
R	density of unparasitised reticulocytes	cells/μl
N	density of unparasitised normocytes	cells/μl
$I_{m,\mathrm{AS}}$	adaptive immune clearance of AS merozoites	s^{-1}
$I_{m,\mathrm{AJ}}$	adaptive immune clearance of AJ merozoites	s^{-1}
$\hat{I}_{m,\mathrm{AS}}$	$I_{m,\mathrm{AS}}/\beta_{N,\mathrm{AS}}$	cells/μl
$\hat{I}_{m,\mathrm{AJ}}$	$I_{m,\mathrm{AJ}}/\beta_{N,\mathrm{AJ}}$	cells/μl
$\lambda_{R,\mathrm{AS}}$	average number of AS parasites per reticulocyte	
$\lambda_{R,\mathrm{AJ}}$	average number of AJ parasites per reticulocyte	
$\lambda_{N,\mathrm{AS}}$	average number of AS parasites per normocyte	
$\lambda_{N,\mathrm{AJ}}$	average number of AJ parasites per normocyte	
α_R	$\exp(-\lambda_{R,\mathrm{AS}} - \lambda_{R,\mathrm{AJ}})$	
α_N	$\exp(-\lambda_{N,\mathrm{AS}} - \lambda_{N,\mathrm{AJ}})$	
**RBC turnover phase variables**		
$r_u(a, t)$	density of unparasitised reticulocytes of age a	cells/μl/day
$N_u(t)$	density of unparasitised normocytes	cells/μl
$R_u(t)$	density of unparasitised reticulocytes	cells/μl
U	uRBC density at start of turnover phase	cells/μl
$P_{s\mathrm{AS}}(t)$	density of singly AS pRBCs	cells/μl
$P_{s\mathrm{AJ}}(t)$	density of singly AJ pRBCs	cells/μl
$P_{m\mathrm{AS}}(t)$	density of multiply AS pRBCs	cells/μl
$P_{m\mathrm{AJ}}(t)$	density of multiply AJ pRBCs	cells/μl
$P_{\mathrm{both}}(t)$	density of multiply AS and AJ pRBCs	cells/μl
$p_{s\mathrm{AS}}(t)$	blood density of AS parasite in singly AS pRBCs	parasites/μl
$p_{s\mathrm{AJ}}(t)$	blood density of AJ parasite in singly AJ pRBCs	parasites/μl
$p_{m\mathrm{AS}}(t)$	blood density of AS parasite in multiply AS pRBCs	parasites/μl
$p_{m\mathrm{AJ}}(t)$	blood density of AJ parasite in multiply AJ pRBCs	parasites/μl
$p_{\mathrm{both,AS}}(t)$	blood density of AS parasite in multiply AS and AJ pRBCs	parasites/μl
$p_{\mathrm{both,AJ}}(t)$	blood density of AJ parasite in multiply AS and AJ pRBCs	parasites/μl
$I_{p,\mathrm{AS}}$	adaptive immune clearance of AS pRBCs	day^{-1}
$I_{p,\mathrm{AJ}}$	adaptive immune clearance of AJ pRBCs	day^{-1}
$I_{p,\mathrm{both}}$	adaptive immune clearance of AS and AJ pRBCs	day^{-1}
I_u	bystander clearance rate of uRBCs	day^{-1}

Table S1: Model variables. Dependence on day i is dropped for clarity.
