# Supplementary material for: Immune-Mediated Competition in Rodent Malaria Is Most Likely Caused by Induced Changes in Innate Immune Clearance of Merozoites
Source: PLoS Comput Biol. 2014 Jan 23;10(1):e1003416. doi: 10.1371/journal.pcbi.1003416 (PMC3900382; doi:10.1371/journal.pcbi.1003416)
Supplement: Table S2 — Model parameters. 1Dependence on AS and AJ removed for brevity. 2 NT is a Normal distribution truncated at 0. (PDF) [file pcbi.1003416.s013.pdf]

Parameter	Description	Units	Prior
**Clone-specific parameters**[1]			
ω	burst size of pRBCs	parasites	$\mathrm{N_T}(7,\ 0.5^2)^2$
P_0	initial parasite density	parasites/μl	$\log P_0 \sim \mathrm{N}(1.5,\ 0.5^2)$
β_R	infection rate of reticulocytes	μl/cell·s	
β_N	infection rate of normocytes	μl/cell·s	
ρ	β_R/β_N		$\log\rho \sim \mathrm{N}(0, 1)$
κ	parasite death rate within multiply parasitised RBCs	day^{-1}	Exp(1)
μ	background loss rate of merozoites	s^{-1}	
$\hat{\mu}$	μ/β_N	cells/μl	Exp(5×10^6)
ν	background loss rate of pRBCs	day^{-1}	Exp(1)
c_m	maximum adaptive immune clearance rate of merozoites	s^{-1}	
$\hat{c}_m$	c_m/β_N	cells/μl	Exp(10^8)
c_p	maximum adaptive immune clearance rate of pRBCs	day^{-1}	Exp(1)
d_m	duration of adaptive immune clearance of merozoites	day	U(0, 20)
d_p	duration of adaptive immune clearance of pRBCs	day	U(0, 20)
	Piecewise linear adaptive immune response		
s_m	start day of clearance of merozoites	day	U(5, 20)
s_p	start day of clearance of pRBCs	day	U(5, 20)
r_m	rise time of clearance of merozoites	day	U(1, 20)
r_p	rise time of clearance of pRBCs	day	U(1, 20)
	Exponential adaptive immune response		
s_m	first day of maximum clearance of merozoites	day	U(5, 20)
s_p	first day of maximum clearance of pRBCs	day	U(5, 20)
	Sigmoidal adaptive immune response		
s_m	day of half maximum clearance of merozoites	day	U(5, 20)
s_p	day of half maximum clearance of pRBCs	day	U(5, 20)
r_m	rate of increase of clearance of merozoites	day^{-1}	Exp(1)
r_p	rate of increase of clearance of pRBCs	day^{-1}	Exp(1)
**Clone-non-specific parameters**			
A	maturation age of reticulocytes	day	2
θ	rate of up-regulation of erythropoiesis	day^{-1}	$\mathrm{N_T}(0.3,\ 0.1^2)$
τ	time lag in erythropoiesis	day	U(0, 6)
K	normal RBC density	cells/μl	$\mathrm{N_T}(10^7,\ 10^{12})$
d	natural death rate of RBCs	day^{-1}	0.025
δ_m	increased death rate of multiply parasitised RBCs	day^{-1}	Exp(1)
ν_{both}	$\frac{\nu_{\mathrm{AS}}+\nu_{\mathrm{AJ}}}{2}$, loss of AJ+AS pRBCs	day^{-1}	
c_u	maximum bystander clearance rate of uRBCs	day^{-1}	Exp(1)
s_u	start day of bystander clearance of uRBCs	day	U(0, 20)
r_u	rise time of bystander clearance of uRBCs	day	U(1, 20)
d_u	duration of bystander clearance of uRBCs	day	U(0, 20)

Table S2: Model parameters. [1]Dependence on AS and AJ removed for brevity. [2]$\mathrm{N_T}$ is a Normal distribution truncated at 0.
