# Supplementary material for: Immune-Mediated Competition in Rodent Malaria Is Most Likely Caused by Induced Changes in Innate Immune Clearance of Merozoites
Source: PLoS Comput Biol. 2014 Jan 23;10(1):e1003416. doi: 10.1371/journal.pcbi.1003416 (PMC3900382; doi:10.1371/journal.pcbi.1003416)
Supplement: Text S1 — Models and methods. A detail description of the various models in the paper and the methods used to compare them. (PDF) [file pcbi.1003416.s014.pdf]

1 Supplementary Material

1.1 The mathematical model

In *Plasmodium chabaudi*, parasitised RBCs (pRBCs) rupture synchronously every 24 hours [27], releasing on average 6-8 parasites (merozoites) into the bloodstream [28]. These newly released merozoites infect further RBCs and the cycle repeats. The rupture of pRBCs (schizogony) occurs at approximately midnight [27, 29].

We use a discrete time formulation to model the dynamics, where each time step corresponds to a single day. The start of day i is defined as the point immediately following rupture of pRBCs, before any infection has occurred (i.e., the point at which merozoites are released into the bloodstream).

We assume that the processes determining RBC density occur on two non-overlapping timescales. The first corresponds to the short infection phase during which merozoites infect RBCs, which occurs within a few minutes following schizogony. The second and subsequent timescale (the remainder of the day) corresponds to the RBC turnover phase: the parasites replicate within pRBCs and new unparasitised RBCs (uRBCs) are released into the bloodstream from the bone marrow. At the end of the RBC turnover phase, surviving pRBCs rupture and release new merozoites.

The model schematic in Fig. S1 shows the biological processes over a 24 hr period included in our models. Tables S1 and S2 list all the variables and parameters used in the model respectively. The prior distributions were based on a previous study of single-clone infections [25].

1.1.1 The infection phase

Some rodent malaria species, such as *P. berghei*, are known to preferentially infect young (1-2 day old), immature RBCs called reticulocytes [47, 48]. By contrast, there is only circumstantial evidence from modelling studies that suggest that *P. chabaudi* prefers reticulocytes [50]. Other models have found no evidence [23]. As discussed in the main text, one possible cause of competition is differential RBC-age preference between the two clones [35, 36]. We therefore want to model RBC age structure. We partition RBCs into reticulocytes and normocytes and allow for different infections rates of each as described in detail below.

The infection dynamics at the start of day i are modelled in continuous time, using a formulation based on [22]. Just after bursting there are populations of free AS and AJ merozoites in the bloodstream with densities $M_{\mathrm{AS},i}$ and $M_{\mathrm{AJ},i}$ respectively. Bursting kills all pRBCs leaving only uRBCs. Let $r_i(a)$ denote the density of unparasitised reticulocytes of age a. Let $R_i = \int_0^A r_i(a)\mathrm{d}a$ denote the total density of unparasitised reticulocytes where A is the maturation age of reticulocytes into normocytes (roughly 1-2 days) and let N_i denote the density of unparasitised normocytes. As infection occurs rapidly over a matter of minutes we can assume that the total reticulocyte and normocyte populations remain constant during infection.

We assume that infection of RBCs by merozoites is density dependent. We also assume that RBCs can be multiply parasitised and that the probability of infection is independent of the number of previous infections. This means we need to keep track of RBCs parasitised by only the AS clone, only the AJ clone and by both clones. We assume AS and AJ merozoites infect reticulocytes at per-capita rates $\beta_{R,\mathrm{AS}}$ and $\beta_{R,\mathrm{AJ}}$ respectively and infect normocytes at rates $\beta_{N,\mathrm{AS}}$ and $\beta_{N,\mathrm{AJ}}$ respectively.

Not all merozoites will invade an RBC, however. Merozoites have been shown to lose infection viability *in vitro* within about 30mins [49]. They are also cleared by the immune response [37, 38]. Following previous models [22, 23], we assume that AS and AJ merozoites are removed from a viable pool at per-capita rates μ_{AS} and μ_{AJ} respectively. These rates combine physical removal,

such as immune-mediated killing, and loss of infection viability.

We also explicitly model adaptive immune responses that develop during the course of infection. We assume that these responses clear AS and AJ merozoites at time-dependent per-capita rates $I_{m,\mathrm{AS},i}$ and $I_{m,\mathrm{AJ},i}$ respectively (see below for the full definitions). Although these adaptive immune responses change over time they will do so on a much longer time scale than the infection phase. Thus we can assume that they are constant over the short infection phases.

The functional forms of the adaptive immune clearance rates are unknown. We therefore require forms that are flexible but minimally parameterised. We tried three forms: piecewise linear, exponential and sigmoidal. The piecewise linear form [25] is defined in terms of four parameters: the day of initial activation s_m, the maximum clearance rate $\hat{c}_m$, the time (measured in days) taken to reach maximum clearance rate r_m, and the duration of the immune response, in days, d_m. Initially, the clearance rates are set to zero. When an immune response is activated, the clearance rate increases to its maximum level over a number of days. Once the given duration has elapsed, as measured from the start day, the clearance rate returns to zero. The expressions for the adaptive immune responses on day i are given by

$$\hat{I}_{m,x,i} = \begin{cases} 0 & \text{if } i < s_{m,x} \\ \hat{c}_{m,x}[i - s_{m,x}]/r_{m,x} & \text{if } s_{m,x} \leq i < s_{m,x} + r_{m,x} \\ \hat{c}_{m,x} & \text{if } s_{m,x} + r_{m,x} \leq i < s_{m,x} + d_{m,x} \\ 0 & \text{if } s_{m,x} + d_{m,x} \leq i \end{cases} \tag{1}$$

where $x \in \{\mathrm{AS}, \mathrm{AJ}\}$.

The exponential form is defined in terms of three parameters: the day on reaching maximum clearance rate s_m, the maximum clearance rate $\hat{c}_m$ and the duration of the response d_m. The clearance rate is 0 on day 0, it then increases exponentially over s_m days up to its maximum $\hat{c}_m$ where it stays until returning to zero after d_m days. The clearance rates are given by the expressions

$$\hat{I}_{m,x,i} = \begin{cases} \exp\left(\ln(\hat{c}_{m,x}+1)i/s_{m,x}\right) - 1 & \text{if } i < s_{m,x} \\ \hat{c}_{m,x} & \text{if } s_{m,x} \leq i < d_{m,x} \\ 0 & \text{if } d_{m,x} \leq i \end{cases} \tag{2}$$

The sigmoidal form is defined in terms of four parameters: the approximate day of half maximum clearance rate s_m, the maximum clearance rate $\hat{c}_m$, the approximate rate of increase on the day of half maximum r_m, and the duration of the response d_m. The clearance rate is 0 on day 0, it then increases sigmoidally, asymptotically approaching its maximum $\hat{c}_m$ until returning to zero after d_m days. The clearance rates are given by the expressions

$$\hat{I}_{m,x,i} = \begin{cases} \hat{c}_{m,x}\frac{1-\exp(-r_{m,x}i)}{1+\exp(-r_{m,x}[i-s_{m,x}])} & \text{if } i < d_{m,x} \\ 0 & \text{if } d_{m,x} \leq i \end{cases} \tag{3}$$

Assuming a well-mixed system with mass-action kinetics, infection of RBCs by merozoites is a Poisson process. This immediately implies that the probability of an event occurring to a merozoite is the rate of that event divided by the sum of the rates of all possible events. For example, the probability of an AS merozoite successfully infecting a normocyte (which occurs at rate $\beta_{N,\mathrm{AS}}N_i$) is $\beta_{N,\mathrm{AS}}N_i/(\beta_{R,\mathrm{AS}}R_i + \beta_{N,\mathrm{AS}}N_i + \mu_{\mathrm{AS}} + I_{m,\mathrm{AS},i})$, where the denominator is the sum of the rates of infecting a reticulocyte, infecting a normocyte, loss of viability and immune clearance. Dividing through by $\beta_{N,\mathrm{AS}}$ and letting $\rho_{\mathrm{AS}} = \beta_{R,\mathrm{AS}}/\beta_{N,\mathrm{AS}}$, $\hat{\mu}_{\mathrm{AS}} = \mu_{\mathrm{AS}}/\beta_{N,\mathrm{AS}}$ and $\hat{I}_{m,\mathrm{AS},i} = I_{m,\mathrm{AS},i}/\beta_{N,\mathrm{AS}}$ results in the probability $N_i/(\rho_{\mathrm{AS}}R_i + N_i + \hat{\mu}_{\mathrm{AS}} + \hat{I}_{m,\mathrm{AS},i})$. Similar probabilities exist for

infection of reticulocytes and for AJ merozoites. This derivation highlights the non-identifiability of (i) the reticulocyte and normocyte infection rates, (ii) the background loss rate of merozoites and the normocyte infection rate and (iii) the adaptive immune clearance rate of merozoites and the normocyte infection rate for each clone. This means we cannot separately estimate these parameters; only their ratios are estimatable.

The parameter $\hat{\mu}$ can be interpreted as the reticulocyte density, weighted by ρ (the ratio of reticulocyte invasion rate to normocyte invasion rate), plus the normocyte density at which a single merozoite has a 50% chance of infecting a RBC in the absence of an adaptive immune response against merozoites.

At the start of the infection phase on day i there are $M_{\mathrm{AS},i}$ AS merozoites. Using the above derived probability for an AS merozoite infecting a normocyte, it is simple to show that the average number of surviving AS parasites per normocyte at the end of the infection phase, denoted $\lambda_{N,\mathrm{AS},i}$, is

$$\lambda_{N,\mathrm{AS},i} = \frac{M_{\mathrm{AS},i}}{\rho_{\mathrm{AS}} R_i + N_i + \hat{\mu}_{\mathrm{AS}} + \hat{I}_{m,\mathrm{AS},i}} \tag{4}$$

Similarly the average number of surviving AJ parasites per normocyte is

$$\lambda_{N,\mathrm{AJ},i} = \frac{M_{\mathrm{AJ},i}}{\rho_{\mathrm{AJ}} R_i + N_i + \hat{\mu}_{\mathrm{AJ}} + \hat{I}_{m,\mathrm{AJ},i}} \tag{5}$$

the average number of surviving AS parasites per reticulocyte is

$$\lambda_{R,\mathrm{AS},i} = \frac{\rho_{\mathrm{AS}} M_{\mathrm{AS},i}}{\rho_{\mathrm{AS}} R_i + N_i + \hat{\mu}_{\mathrm{AS}} + \hat{I}_{m,\mathrm{AS},i}} \tag{6}$$

and the average number of surviving AJ parasites per reticulocyte is

$$\lambda_{R,\mathrm{AJ},i} = \frac{\rho_{\mathrm{AJ}} M_{\mathrm{AJ},i}}{\rho_{\mathrm{AJ}} R_i + N_i + \hat{\mu}_{\mathrm{AJ}} + \hat{I}_{m,\mathrm{AJ},i}} \tag{7}$$

Since infections occur independently, the number of parasites in a RBC is Poisson distributed. Thus the probability of a reticulocyte being infected with n_{AS} AS parasites and n_{AJ} AJ parasites is

$$\Pr(n_{\mathrm{AS}}, n_{\mathrm{AJ}}) = \frac{\lambda_{R,\mathrm{AS}}^{n_{\mathrm{AS}}} e^{-\lambda_{R,\mathrm{AS}}}}{n_{\mathrm{AS}}!} \frac{\lambda_{R,\mathrm{AJ}}^{n_{\mathrm{AJ}}} e^{-\lambda_{R,\mathrm{AJ}}}}{n_{\mathrm{AJ}}!} \tag{8}$$

and similarly for a normocyte

$$\Pr(n_{\mathrm{AS}}, n_{\mathrm{AJ}}) = \frac{\lambda_{N,\mathrm{AS}}^{n_{\mathrm{AS}}} e^{-\lambda_{N,\mathrm{AS}}}}{n_{\mathrm{AS}}!} \frac{\lambda_{N,\mathrm{AJ}}^{n_{\mathrm{AJ}}} e^{-\lambda_{N,\mathrm{AJ}}}}{n_{\mathrm{AJ}}!} \tag{9}$$

From these equations we can derive the density of unparasitised reticulocytes and normocytes by setting $n_{\mathrm{AS}} = 0$ and $n_{\mathrm{AJ}} = 0$, singly parasitised RBCs by setting either $n_{\mathrm{AS}} = 1$ and $n_{\mathrm{AJ}} = 0$ or $n_{\mathrm{AS}} = 0$ and $n_{\mathrm{AJ}} = 1$, and multiply parasitised RBCs by setting $n_{\mathrm{AS}} > 1$ and $n_{\mathrm{AJ}} = 0$ or $n_{\mathrm{AS}} = 0$ and $n_{\mathrm{AJ}} > 1$ or $n_{\mathrm{AS}} > 0$ and $n_{\mathrm{AJ}} > 0$ and summing terms. Note, we do not need to separately keep track of parasitised reticulocytes and normocytes as they are not measured and they die after 24 hours. We can also derive the total blood density of parasites at the end of the infection phase

by multiplying the above probabilities by the number of parasites of each strain in a RBC, i.e., $n_{\mathrm{AS}} n_{\mathrm{AJ}} \Pr(n_{\mathrm{AS}}, n_{\mathrm{AJ}})$.

These densities are given as follows. First, let $\alpha_R = \exp(-\lambda_{R,\mathrm{AS}} - \lambda_{R,\mathrm{AJ}})$ and $\alpha_N = \exp(-\lambda_{N,\mathrm{AS}} - \lambda_{N,\mathrm{AJ}})$. Then the density of unparasitised reticulocytes of age a on day i at the end of the infection phase is (and dropping the dependence on i for clarity)

$$r_u(a,0) = \alpha_R r(a) \text{ for all } a \in [0, A] \tag{10}$$

the density of unparasitised normocytes is

$$N_u(0) = \alpha_N N \tag{11}$$

the densities of RBCs (reticulocytes plus normocytes) parasitised by a single AS or AJ parasite are

$$P_{s\mathrm{AS}}(0) = \alpha_R \lambda_{R,\mathrm{AS}} R + \alpha_N \lambda_{N,\mathrm{AS}} N \tag{12}$$
$$P_{s\mathrm{AJ}}(0) = \alpha_R \lambda_{R,\mathrm{AJ}} R + \alpha_N \lambda_{N,\mathrm{AJ}} N \tag{13}$$

the densities of RBCs parasitised by multiple AS or AJ parasites are

$$P_{m\mathrm{AS}}(0) = \alpha_R [e^{\lambda_{R,\mathrm{AS}}} - 1 - \lambda_{R,\mathrm{AS}}] R + \alpha_N [e^{\lambda_{N,\mathrm{AS}}} - 1 - \lambda_{N,\mathrm{AS}}] N \tag{14}$$
$$P_{m\mathrm{AJ}}(0) = \alpha_R [e^{\lambda_{R,\mathrm{AJ}}} - 1 - \lambda_{R,\mathrm{AJ}}] R + \alpha_N [e^{\lambda_{N,\mathrm{AJ}}} - 1 - \lambda_{N,\mathrm{AJ}}] N \tag{15}$$

the density of RBCs parasitised by multiple AS and AJ parasites is

$$P_{\mathrm{both}}(0) = \alpha_R [e^{\lambda_{R,\mathrm{AS}}} - 1][e^{\lambda_{R,\mathrm{AJ}}} - 1] R + \alpha_N [e^{\lambda_{N,\mathrm{AS}}} - 1][e^{\lambda_{N,\mathrm{AJ}}} - 1] N \tag{16}$$

the blood densities of AS and AJ parasites in single parasitised RBCs are

$$p_{s\mathrm{AS}}(0) = P_{s\mathrm{AS}}(0) \tag{17}$$
$$p_{s\mathrm{AJ}}(0) = P_{s\mathrm{AJ}}(0) \tag{18}$$

the blood densities of AS and AJ parasites in RBCs multiply parasitised by AS or AJ parasites are

$$p_{m\mathrm{AS}}(0) = \alpha_R \lambda_{R,\mathrm{AS}} [e^{\lambda_{R,\mathrm{AS}}} - 1] R + \alpha_N \lambda_{N,\mathrm{AS}} [e^{\lambda_{N,\mathrm{AS}}} - 1] N \tag{19}$$
$$p_{m\mathrm{AJ}}(0) = \alpha_R \lambda_{R,\mathrm{AJ}} [e^{\lambda_{R,\mathrm{AJ}}} - 1] R + \alpha_N \lambda_{N,\mathrm{AJ}} [e^{\lambda_{N,\mathrm{AJ}}} - 1] N \tag{20}$$

and the blood densities of AS and AJ parasites in RBCs multiply parasitised by AS and AJ parasites are

$$p_{\mathrm{both,AS}}(0) = \lambda_{R,\mathrm{AS}} [1 - e^{-\lambda_{R,\mathrm{AS}}}] R + \lambda_{N,\mathrm{AS}} [1 - e^{-\lambda_{N,\mathrm{AS}}}] N \tag{21}$$
$$p_{\mathrm{both,AJ}}(0) = \lambda_{R,\mathrm{AJ}} [1 - e^{-\lambda_{R,\mathrm{AJ}}}] R + \lambda_{N,\mathrm{AJ}} [1 - e^{-\lambda_{N,\mathrm{AJ}}}] N \tag{22}$$

We also define the total uRBC density at the end of the infection phase as $U = N_u + \int_0^A r_u(a,0)\,\mathrm{d}a$. All these densities form the initial conditions of the subsequent RBC turnover phase.

1.1.2 RBC turnover phase

The RBC turnover dynamics occur after the infection dynamics on each day i. They are modelled in continuous time $t \in [0, 1]$, which has units of days.

In the absence of infection, RBCs are lost through natural mortality and gained through the production of new cells (called erythropoiesis). We take natural RBC decay rate as $d = 0.025\,\text{day}^{-1}$ [30, 31, 24].

Erythropoiesis is up-regulated in response to the anaemia caused by infection and rupture [33, 34, 22, 25]. We assume that the level of up-regulation on day i is linearly proportional to the difference between the normal RBC density, K, and the uRBC density τ days earlier, $U_{i-\tau}$. The parameter τ measures the feedback lag (in days) between RBC density and the level of erythropoiesis. (More complicated functional forms have been used in other modelling studies [36, 40]. However, we have found this simple linear relationship to adequately explain RBC dynamics in the acute phase.) The up-regulation of erythropoiesis is then given by $\theta[K - U_{i-\tau}]$, where θ is the proportion of the RBC deficit that is recovered in a single day.

Within-RBC interference competition between parasites is modelled as an additional mortality of parasites. Let κ_{AS} and κ_{AJ} be the interference-induced mortality rates of AS and AJ parasites, respectively, in multiply parasitised RBCs.

We assume constant background mortality rates ν_{AS}, ν_{AJ} and ν_{both} of pRBCs infected with only the AS clone, only the AJ clone and with both clones respectively. We also assume that multiply-parasitised RBCs have an additional mortality rate, δ_m. This allows for the possibility that these RBCs may be exploited more rapidly than singly-parasitised cells and die before schizogony.

As for merozoites, we explicitly model adaptive immune responses that clear pRBCs. We assume that these responses clear pRBCs at time-dependent per-capita rates $I_{p,\text{AS},i}$, $I_{p,\text{AJ},i}$ and $I_{p,\text{both},i}$ for AS pRBCs, AJ pRBCs and AS plus AJ pRBCs respectively. As for merozoites, we examined three forms for these clearance rates: piecewise linear, exponential and sigmoidal. The expressions are similar to those for merozoites above. In addition, we assume that pRBCs infected by both AS and AJ clones are cleared at the maximum of the rates for single-clone infected RBCs, i.e., $I_{p,\text{both},i} = \max(I_{p,\text{AS},i}, I_{p,\text{AJ},i})$.

It has been demonstrated that uRBCs can also be removed in a process called bystander killing [39]. This is modelled as a time-dependent (piecewise linear) per-capita clearance rate $I_{u,i}$ of uRBCs [25]:

$$I_{u,i} = \begin{cases} 0 & \text{if } i < s_u \\ c_u[i - s_u]/r_u & \text{if } s_u \le i < s_u + r_u \\ c_u & \text{if } s_u + r_u \le i < s_u + d_u \\ 0 & \text{if } s_u + d_u \le i \end{cases} \tag{23}$$

Given the above assumptions, the dynamics of the RBC turnover phase are described by the following system of differential equations (dependence on i is dropped for clarity): for uninfected reticulocyte and normocyte densities

$$\frac{\partial r_u(a,t)}{\partial t} + \frac{\partial r_u(a,t)}{\partial a} = -[d + I_u + \delta(a - A)]r_u(a,t) \text{ for all } a \in [0, A] \tag{24}$$

$$\frac{\mathrm{d}N_u(t)}{\mathrm{d}t} = -[d + I_u]N_u(t) + \delta(a - A)r_u(a,t) \tag{25}$$

where $\delta(\cdot)$ is the Dirac-delta function. For pRBC densities

$$\frac{\mathrm{d}P_{s\mathrm{AS}}(t)}{\mathrm{d}t} = -[d + \nu_{\mathrm{AS}} + I_{p,\mathrm{AS}}]P_{s\mathrm{AS}}(t) \tag{26}$$

$$\frac{\mathrm{d}P_{s\mathrm{AJ}}(t)}{\mathrm{d}t} = -[d + \nu_{\mathrm{AJ}} + I_{p,\mathrm{AJ}}]P_{s\mathrm{AJ}}(t) \tag{27}$$

$$\frac{\mathrm{d}P_{m\mathrm{AS}}(t)}{\mathrm{d}t} = -[d + \nu_{\mathrm{AS}} + I_{p,\mathrm{AS}} + \delta_m]P_{m\mathrm{AS}}(t) \tag{28}$$

$$\frac{\mathrm{d}P_{m\mathrm{AJ}}(t)}{\mathrm{d}t} = -[d + \nu_{\mathrm{AJ}} + I_{p,\mathrm{AJ}} + \delta_m]P_{m\mathrm{AJ}}(t) \tag{29}$$

$$\frac{\mathrm{d}P_{\mathrm{both}}(t)}{\mathrm{d}t} = -[d + \nu_{\mathrm{both}} + I_{p,\mathrm{both}} + \delta_m]P_{\mathrm{both}}(t) \tag{30}$$

and for parasite blood densities

$$\frac{\mathrm{d}p_{s\mathrm{AS}}(t)}{\mathrm{d}t} = -[d + \nu_{\mathrm{AS}} + I_{p,\mathrm{AS}}]p_{s\mathrm{AS}}(t) \tag{31}$$

$$\frac{\mathrm{d}p_{s\mathrm{AJ}}(t)}{\mathrm{d}t} = -[d + \nu_{\mathrm{AJ}} + I_{p,\mathrm{AJ}}]p_{s\mathrm{AJ}}(t) \tag{32}$$

$$\frac{\mathrm{d}p_{m\mathrm{AS}}(t)}{\mathrm{d}t} = -[d + \nu_{\mathrm{AS}} + I_{p,\mathrm{AS}} + \delta_m + \kappa_{\mathrm{AS}}]p_{m\mathrm{AS}}(t) \tag{33}$$

$$\frac{\mathrm{d}p_{m\mathrm{AJ}}(t)}{\mathrm{d}t} = -[d + \nu_{\mathrm{AJ}} + I_{p,\mathrm{AJ}} + \delta_m + \kappa_{\mathrm{AJ}}]p_{m\mathrm{AJ}}(t) \tag{34}$$

$$\frac{\mathrm{d}p_{\mathrm{both,AS}}(t)}{\mathrm{d}t} = -[d + \nu_{\mathrm{both}} + I_{p,\mathrm{both}} + \delta_m + \kappa_{\mathrm{AS}}]p_{\mathrm{both,AS}}(t) \tag{35}$$

$$\frac{\mathrm{d}p_{\mathrm{both,AJ}}(t)}{\mathrm{d}t} = -[d + \nu_{\mathrm{both}} + I_{p,\mathrm{both}} + \delta_m + \kappa_{\mathrm{AJ}}]p_{\mathrm{both,AJ}}(t) \tag{36}$$

The boundary condition, $r_{u,i}(0,t) = dK + \theta[K - U_{i-\tau}]$, for Equation 24 represents migration of new reticulocytes from bone marrow and spleen into the circulation. We assume it is constant throughout the day. This assumption is for computational convenience, otherwise we would have to solve delay differential equations which would become computational prohibitive. Given that upregulation is only important for the RBC dynamics after peak anaemia, this assumption has little consequence for our conclusions about immune-mediated competition.

The initial conditions for Equations 24-36 are obtained from Equations 10-22. The solutions of Equations 24 and 25 are

$$r_{u,i}(a,t) = \begin{cases} [dK + \theta[K - U_{i-\tau}]]\, e^{-[d+I_{u,i}]a} & \text{if } 0 \le a < t \\ r_{u,i}(a-t,0)e^{-[d+I_{u,i}]t} & \text{if } t \le a < A \end{cases} \tag{37}$$

$$N_{u,i}(t) = \left[N_{u,i}(0) + \int_{A-t}^{A} r_{u,i}(a,0)\,\mathrm{d}a\right] e^{-[d+I_{u,i}]t} \tag{38}$$

where $r_{u,i}(a,0)$ is the initial age distribution of unparasitised reticulocyte density on day i and $N_{u,i}(0)$ the initial density of unparasitised normocytes on day i. The solutions of Equations 26-36 are simply decaying exponentials. For example, the solution of Equation 26 is $P_{s\mathrm{AS},i}(t) = P_{s\mathrm{AS},i}(0)\exp(-[d + \nu_{\mathrm{AS}} + I_{p,\mathrm{AS}}]t)$. In order to compute $r_{u,i}(a,t)$ we discretise reticulocyte age a. The maximum discritisation possible is $\Delta a = 8\,\mathrm{hrs}$ as this is the time between bursting at midnight and observation at 8am. We have found that smaller discritisations do not affect model fits or the inferences drawn from them. We therefore choose this discritisation for computational speed.

1.1.3 Rupture of parasitised RBCs

The final event on day i is the rupture of pRBCs and release of free merozoites at midnight. At this point each surviving AS and AJ parasite produces ω_{AS} and ω_{AJ} merozoites, respectively, and all pRBC densities return to zero. We assume the burst sizes are independent of the age of the pRBC. The AS and AJ merozoite densities after rupture on the $(i+1)^{th}$ day are then given by

$$M_{\mathrm{AS},i+1} = \omega_{\mathrm{AS}}[p_{s\mathrm{AS},i}(1) + p_{m\mathrm{AS},i}(1) + p_{\mathrm{both,AS},i}(1)] \tag{39}$$

$$M_{\mathrm{AJ},i+1} = \omega_{\mathrm{AJ}}[p_{s\mathrm{AJ},i}(1) + p_{m\mathrm{AJ},i}(1) + p_{\mathrm{both,AJ},i}(1)] \tag{40}$$

The unparasitised reticulocyte density is $R_{i+1} = R_{u,i}(1)$, and the unparasitised normocyte density is $N_{i+1} = N_{u,i}(1)$, which are needed for Equations 4-7. Also needed is the density of unparasitised reticulocytes of age a for Equation 10, $r_{i+1}(a) = r_{u,i}(a,1)$.

1.1.4 Initial conditions

During the experiments the mice were inoculated intraperitoneally with 10^5 AS pRBCs, 10^5 AJ pRBCs or both at 08:00 hrs on day 0. However, it might be the case that not all parasites enter the bloodstream. We therefore estimate the initial circulating AS and AJ parasite densities, $P_{0,\mathrm{AS}}$ and $P_{0,\mathrm{AJ}}$ from the data. The initial conditions on day 0 are, therefore, $P_{s\mathrm{AS},0}(1/3) = P_{0,\mathrm{AS}}$ or 0, $P_{s\mathrm{AJ},0}(1/3) = P_{0,\mathrm{AJ}}$ or 0, $P_{m\mathrm{AS},0}(1/3) = P_{m\mathrm{AJ},0}(1/3) = P_{\mathrm{both},0}(1/3) = 0$, $p_{s\mathrm{AS},0}(1/3) = P_{0,\mathrm{AS}}$ or 0, $p_{s\mathrm{AJ},0}(1/3) = P_{0,\mathrm{AJ}}$ or 0, $p_{m\mathrm{AS},0}(1/3) = P_{m\mathrm{AJ},0}(1/3) = P_{\mathrm{both,AS},0}(1/3) = P_{\mathrm{both,AJ},0}(1/3) = 0$.

The initial total RBC density in the mice was assumed to be the normal RBC density in the absence of an infection. RBCs die naturally at rate $d = 0.025\mathrm{day}^{-1}$ [32]. The initial total density of RBCs K, is partitioned between the reticulocytes and normocytes as follows

$$r_{u,0}(a, 1/3) = dKe^{-da} \text{ for all } a \in [0, A] \tag{41}$$

$$N_{u,0}(1/3) = Ke^{-dA} \tag{42}$$

K is estimated from the data.

1.2 McMC diagnostics and model adequacy

The convergences of the Markov chains to the posteriors of each mouse are assessed using the Gelman-Rubin statistic [52]. Figure S2 shows the Gelman-Rubin statistics of each parameter of the all-cause model for all mice. A statistic below 1.1 suggests excellent convergence of the Markov chains [51].

The standardised residuals for the all-cause model for the three mice phenotypes are given in (Figs. S3, S4 and S5). The standardised residuals of an adequate model should be approximately normally distributed with mean 0 and standard deviation 1.

The Q-Q plot for the all-cause model for the three mouse phenotypes are given in (Figs. S6, S7 and S8). The overlaid residuals and the Normal Q-Q plot in all three suggest adequate fits with some minor over and under estimation of the dynamics.

1.3 Likelihood plots by mouse phenotypes

Figures S9-S11 show the plots of $\log_{10}$-marginal likelihood against $\log_{10}$-maximum likelihood of the models tested in Table 2 for each mouse phenotype. The ability of nude mice to discriminate between hypotheses is weak. However, regardless of phenotype, hypothesis H_5 is always the minimally adequate model.
